# Inhibiting Glycogen Synthase Kinase 3 Suppresses TDP-43-Mediated Neurotoxicity in a Caspase-Dependent Manner

**DOI:** 10.1007/s12035-026-05675-5

**Published:** 2026-01-17

**Authors:** Matthew Anthony White, Leon Crowley, Francesca Massenzio, Xingli Li, Michael Niblock, Sara Milani, Michael Philip Coleman, Sami J. Barmada, Jemeen Sreedharan

**Affiliations:** 1 Department of Basic and Clinical Neuroscience, The Maurice Wohl Clinical Neuroscience Institute, Institute of Psychiatry, Psychology and Neuroscience (IoPPN), King’s College London, London, SE5 9RT UK; 2 Department of Neurology, Michigan Medicine, University of Michigan, Ann Arbor, MI USA; 3 Neuroscience Graduate Program, Michigan Medicine, University of Michigan, Ann Arbor, MI USA; 4 Cellular and Molecular Biology Program, Michigan Medicine, University of Michigan, Ann Arbor, MI USA; 5 The Babraham Institute, Cambridge, UK; 6 John Van Geest Centre for Brain Repair, University of Cambridge, Cambridge, UK; 7 Present address, : Department of Pharmacy and Biotechnology, University of Bologna, Bologna, Italy

**Keywords:** TDP-43, GSK3 inhibition, ALS-FTD, Neurodegeneration, Caspase-dependent cleavage, Neurotoxicity attenuation, TDP-43 C-terminal fragments, Kinase inhibition

## Abstract

Amyotrophic lateral sclerosis (ALS) and frontotemporal dementia (FTD) are progressive neurodegenerative diseases characterised by TAR DNA-binding protein 43 kDa (TDP-43) pathology. We previously showed that deletion of glycogen synthase kinase-3 (GSK3) suppresses TDP-43-mediated motor neuron degeneration in *Drosophila*. Here, we investigated the potential of GSK3 inhibition to ameliorate TDP-43-mediated toxicity in mammalian neurons. We show that TDP-43 activates GSK3 and promotes caspase-dependent cleavage of TDP-43, generating C-terminal fragments. We determine the functional importance of the N-terminal Asp89 caspase cleavage site in regulating TDP-43 proteostasis in both wild-type and ALS-linked TDP-43 variants and show that GSK3 inhibition selectively reduces truncated TDP-43 species, lowers nuclear TDP-43 levels, and improves neuronal survival. Neuroprotective effects were conserved in primary rodent cortical neurons, primary mouse motor neurons, and human iPSC-derived cortical neurons, highlighting the potentially broad therapeutic potential of GSK3 inhibition. We also find that the GSK3 inhibitor CHIR99021 reduces GSK3 RNA and protein expression and increases GSK3 phosphorylation, indicating novel mechanisms by which it acts to inhibit GSK3 activity. Unexpectedly, an N-terminally truncated variant (TDP-43^N-Del^), originally designed as a negative transfection control, exerted modest toxicity, potentially through retained susceptibility to caspase cleavage. Together, our findings uncover a caspase-mediated mechanism linking GSK3 activity to TDP-43 turnover, localisation, and neurotoxicity, and position GSK3 inhibition as a promising strategy to mitigate TDP-43-driven neurodegeneration in ALS-FTD.

**Supplementary Information:**

The online version contains supplementary material available at https://doi.org/10.1007/s12035-026-05675-5.

## Introduction

Amyotrophic lateral sclerosis (ALS) and frontotemporal dementia (FTD) are progressive and fatal neurodegenerative diseases that exist on a clinicopathological spectrum (ALS-FTD) [1]. Clinically, ALS is characterised by motor dysfunction, while FTD leads to a decline in cognition affecting executive functions, behaviour, and language capabilities. The available disease-modifying drugs have only a minor impact on survival and disease progression, and novel therapeutic agents are therefore urgently required.

Almost all ALS and half of FTD cases are characterised by cytoplasmic ubiquitinated inclusions positive for TAR DNA-binding protein 43 kDa (TDP-43)[2–4]. Disease-linked mutations in *TARDBP* (the gene encoding TDP-43) indicate a fundamental role for TDP-43 in ALS-FTD pathogenesis [5–8]. TDP-43 inclusions are also seen in Alzheimer’s disease [9, 10], Parkinson’s disease [11, 12], Huntington’s disease [13] and limbic-predominant age-related TDP-43 encephalopathy (LATE)[14]. These observations implicate aberrant homeostasis of TDP-43 in a broad range of neurodegenerative diseases.

Caspases [15–18] and calpains [19, 20] can cleave TDP-43 to generate 25, 35, and 42 kDa C-terminal fragments. The accumulation of these phosphorylated and aggregated C-terminal fragments is a hallmark of ALS-FTD [3, 21, 22]. Cleavage products of TDP-43 can be degraded by the proteasome and through autophagy [23–25]. However, whether aggregated and cleaved TDP-43 mediate disease or are non-toxic byproducts of physiological TDP-43 processing is unclear [26, 27].

Glycogen Synthase Kinase-3 (GSK3) is a highly conserved and ubiquitously expressed serine/threonine protein kinase with wide-ranging biological functions including glycogen metabolism, cell proliferation, and apoptosis [28]. Mammalian GSK3 is encoded by two gene paralogues, *GSK3A* and *GSK3B*, which give rise to two protein isoforms, GSK3α and GSK3β. Several lines of study link GSK3 biology to ALS-FTD pathogenesis. Firstly, expression of GSK3 is significantly increased in thoracic spinal cord tissue of patients with apparently sporadic ALS [29] and increased expression of GSK3 isoform β can be seen in frontal, hippocampal, cerebellar, cervical, and lumbar tissue of patients with ALS or ALS with cognitive impairment [30]. Secondly, TDP-43 expression induces GSK3 [31] whose activity modulates ER-mitochondrial associations regulated by vesicle-associated membrane protein-associated protein-B [32]. Thirdly, GSK3 is a modulator of TDP-43 cytosolic accumulation during cellular stress, and its inhibition reduces the cytosolic accumulation of C-terminal TDP-43 fragments [33]. Finally, in an unbiased in vivo screen, we previously showed that deletion of *shaggy*, the *Drosophila* orthologue of GSK3, significantly suppresses TDP-43-induced motor axon and neuromuscular junction degeneration [34]. Collectively, these data suggest that increased GSK3 activity plays a key role in neurodegeneration associated with TDP-43. Here, using overexpression models of wild-type and mutant TDP-43, we confirm that inhibition of GSK3α and GSK3β mitigates TDP-43-linked neurodegeneration in rodent primary and human iPSC-derived neurons, and explore the role of caspase cleavage of TDP-43 in this process.

## Materials and Methods

### Mouse Breeding and Maintenance

This study was conducted on tissues from wild-type C57Bl/6J mice (*Mus musculus*) and rats (*Rattus norvegicus*) with breeding carried out in the UK and USA. All rodent work in the UK was conducted in accordance with the United Kingdom Animals (Scientific Procedures) Act (1986) and the United Kingdom Animals (Scientific Procedures) Act (1986) Amendment Regulations 2012. Experiments in the USA were approved by the Committee on the Use and Care of Animals (UCUCA) at the University of Michigan and performed in accordance with UCUCA guidelines. Mice were housed in cages of up to five animals under a 12 h light/dark cycle, and rats were housed singly in chambers equipped with environmental enrichment.

### Plasmid Constructs and Small Molecule Compounds

The GFP-tagged TDP-43 expression constructs TDP-43^WT^ and TDP-43^Q331K^ were adapted from previously generated plasmids [6] by amplification of the TDP-43 open reading frame and ligation into the pEGFP-N1 vector. TDP-43^N-Del^ was generated by deletion of the first 81 amino acids from the N-terminus of TDP-43^WT^ using the QuickChange Site-Directed Mutagenesis Kit (Agilent). GSK3 expression constructs were obtained from Addgene: pcDNA5FRT PUR mGSK3AWT was a gift from Jim Woodgett (Addgene plasmid #35159; http://n2t.net/addgene:35159; RRID:Addgene_35159); Tag5Amyc-GSK3b KD was a gift from Mien-Chie Hung (Addgene plasmid #16262; http://n2t.net/addgene:16262; RRID:Addgene_16262).

The GSK3 inhibitor CHIR99021, CAS: 252917-06−9, was obtained from Abcam (ab120890) and a 100 µM stock in DMSO stored at −20 °C. AZD 1080, CAS: 612487-72−6 was kindly provided by Dr. Richard Mead, reconstituted in DMSO and stored at −80 °C until use. The cell-permeable, irreversible caspase inhibitor Q-VD-OPh and broad-spectrum protein kinase inhibitor staurosporine were both reconstituted in DMSO and stored at −20 °C.

### SH-SY5Y Cell Line Culture

SH-SY5Y cells were maintained in DMEM/F-12, supplemented with GlutaMAX™ (Gibco, Thermo Fisher Scientific), 10% fetal bovine serum (FBS) (Gibco, Thermo Fisher Scientific), 1% Penicillin-Streptomycin (10,000 U/ml, Thermo Fisher Scientific), and maintained at 37 °C in a humidified 5% CO_2_ incubator.

### SH-SY5Y Cell Transfection and Treatment

For western blots, cells were passaged, plated, and allowed to recover for 24 h. Cells were transiently transfected with plasmid constructs expressing GFP or GFP-tagged TDP-43^N-Del^, TDP-43^WT^, or TDP-43^Q331K^ with TurboFect™ Transfection Reagent (Thermo Fisher Scientific) according to the manufacturer’s protocol. For cells treated with the small molecule GSK3 inhibitors, CHIR99021 or AZD1080, and the pan-caspase inhibitor, Q-VD-OPh, compounds were administered at the time of transfection. After 24 h, cells were lysed in RIPA buffer containing 10 µg/ml protease and phosphatase inhibitor cocktail (Merck). For experiments using the pro-apoptotic caspase activator staurosporine, this was administered 3 h before sample collection in RIPA buffer unless otherwise stated. Lysates were cleared by centrifugation and stored at −20 °C until use. Transfection efficiency was not systematically quantified across all assays, but based on visual inspection of GFP fluorescence, we consistently observed robust expression in a high proportion of cells (typically > 50%) under the conditions used. No substantial differences in apparent transfection efficiency were noted between constructs.

For fluorescence imaging, cells were passaged and plated at a density of 1.5 × 10^4^ cells/well in CellCarrier-96 Ultra Microplates (Perkin Elmer) previously coated with Poly-DL-ornithine hydrobromide 100 mg (0.5 mg/ml; Sigma). After 24 h in culture, cells were transiently transfected in the same manner as for western blot experiments.

### TDP-43 Knockdown

For siRNA knockdown experiments, 3 × 10^5^ SH-SY5Y cells were reverse-transfected in six-well plates using RNAiMAX (Thermo Fisher Scientific) and a human *TARDBP* SMARTpool ON-TARGETplus siRNA (Horizon Discovery) according to the manufacturer’s protocol. BLOCK-iT™ Alexa Fluor™ Red Fluorescent Control siRNA (Thermo Fisher Scientific) was used to control for non-specific effects of siRNA transfection. Culture medium was changed the following day, and cells were collected in RIPA lysis buffer (Thermo Fisher Scientific) supplemented with 10% Halt™ Protease and Phosphatase Inhibitor Cocktail (Thermo Fisher Scientific) at 72 h post-transfection unless otherwise stated.

### qPCR Following GSK3 Inhibition

For quantitative PCR experiments, SH-SY5Y cells were treated with 10 µM CHIR99021 for 24 h in six-well plates. Cells were harvested in RLT buffer (Qiagen) supplemented as per the kit instructions, and RNA was processed according to the manufacturer’s protocol. RNA (500 ng) was reverse transcribed (QuantiTect Reverse Transcription Kit, Qiagen) and the output volume of 20 µl diluted tenfold in nuclease-free water (Promega). Real-time PCR was performed using specific primers (KiCqStart SYBR Green, Merck) and PowerUp SYBR Master Mix (Thermo Fisher Scientific) on a QuantStudio 6 Flex instrument using fast cycling mode based on the user guide (publication number MAN0013511 Rev. F.0, Applied Biosystems). Reaction specificity was confirmed by melt curve analysis and normalised expression (ΔΔCq) calculated manually using three reference genes: GAPDH, ACTB, and YWHAZ.

### Western Blot Analysis (for Uncropped Western Blot Images, See Supplementary Fig. 6)

**Fig. 1:**
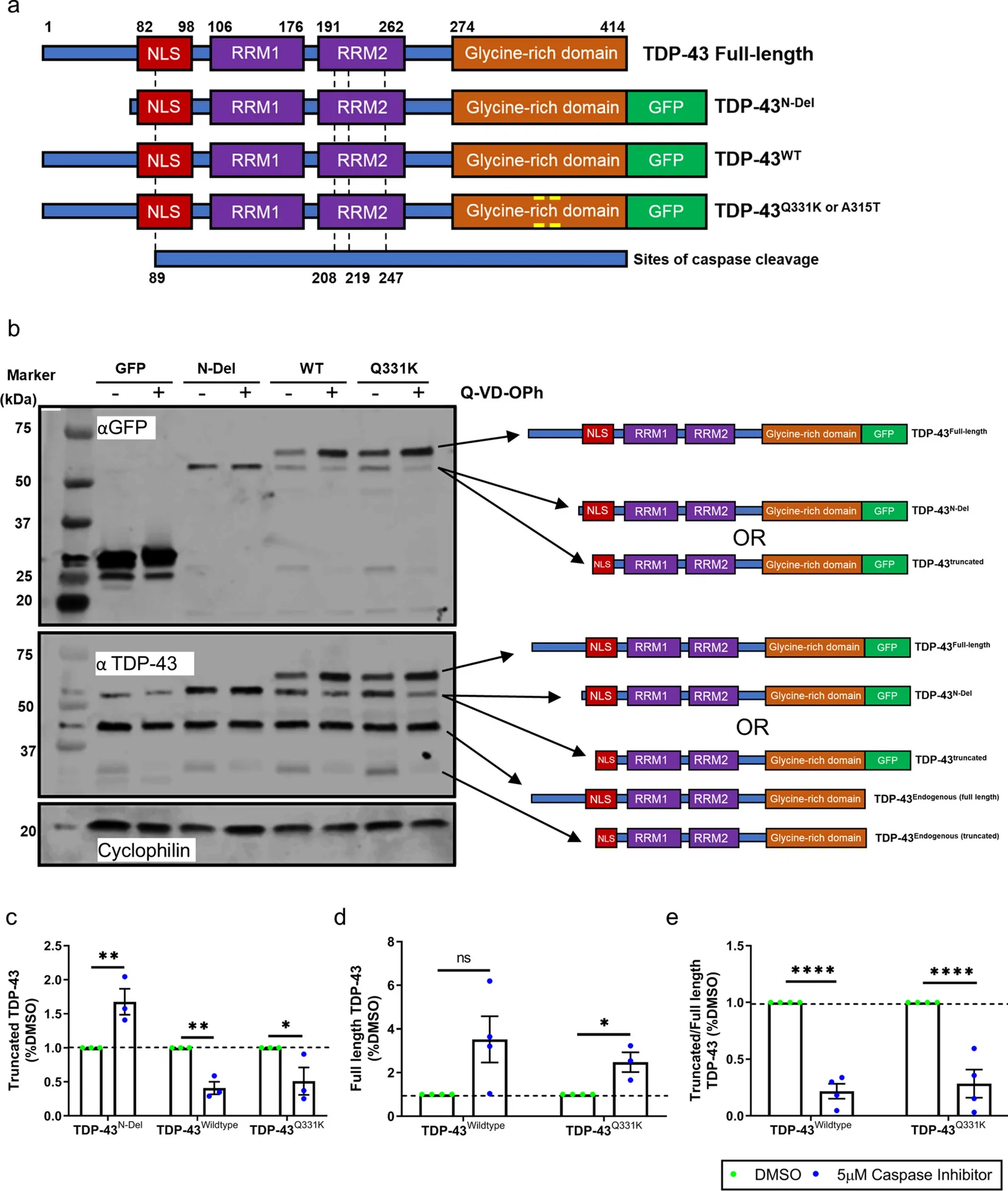
TDP-43 undergoes caspase-mediated cleavage. **a** Schematic of GFP-tagged TDP-43 expression constructs used in this study. The dotted lines indicate predicted sites of caspase-mediated cleavage. TDP-43^N-Del^ lacks amino acids 1–81, removing the N-terminal dimerisation domain but retaining the Asp89 caspase recognition site within the nuclear localisation signal (NLS). **b** Representative immunoblot of SH-SY5Y cells transfected for 24 h with GFP alone or GFP-tagged TDP-43^N-Del^, TDP-43^WT^, or TDP-43^Q331K^, with or without the pan-caspase inhibitor Q-VD-OPh. **c**–**e** Quantification of GFP-tagged TDP-43 immunoblot bands, showing the abundance of truncated species (**c**), full-length species (**d**), and the truncated:full-length ratio (**e**) in the absence or presence of caspase activity (DMSO vs. Q-VD-OPh). Pairwise comparisons: Truncated TDP-43 (**c**): ** (*P* = 0.001679) for TDP-43^N-Del^; ** (*P* = 0.004268) for TDP-43^WT^; * (*P* = 0.013091) for TDP-43^Q331K^. Full-length TDP-43 (**d**): ns (*P* = 0.054872) for TDP-43^WT^; * (*P* = 0.011711) for TDP-43^Q331K^. For (**c**–**e**): *n* = 3–4 biological replicates per condition; ****P < 0.0001; multiple t-test with Holm–Sidak correction. Error bars denote mean ± s.e.m

Protein lysates from SH-SY5Y cells were quantified (bicinchoninic acid protein assay, Pierce) and then electrophoresed in 4–12% Mini-PROTEAN® TGX™ Precast Gels (Bio-Rad) in 1X TGS Tris/Glycine/SDS Buffer (10X TGS Buffer, Bio-Rad). Gels were wet transferred to PVDF membranes (Millipore), blocked with a 50:50 mixture of Odyssey PBS blocking buffer (Li-Cor) and PBS with 0.1% Tween-20 for 1 h at room temperature, and then probed with primary antibodies (Supplementary Table 1) at 4 °C overnight, diluted 1:1000 in PBS Tween-20 (Sigma). For Fig. 1 and Fig. 4, endogenous TDP-43 was labelled using the mouse monoclonal antibody ab57105, while for Supplementary Fig. 4, endogenous TDP-43 was labelled using the rabbit polyclonal antibody 10782-2-AP. For primary antibodies recognising phosphorylated GSK3, an alternative TBS blocking buffer (Li-Cor) was used. Membranes were subsequently probed for 1 h at room temperature with the following secondary antibodies: IRDye 680RD goat anti-Mouse and IRDye 800CW Donkey anti-rabbit (Li-Cor). Secondary antibodies were fluorescently tagged for Odyssey imaging. Membranes were scanned on an Odyssey® CLx Imaging scanner (Li-Cor) and quantified using Image Studio Lite software.

### High Content Imaging

To quantify nuclear TDP-43 intensity in SH-SY5Y cells expressing GFP-tagged TDP-43 constructs, cells were imaged using an Opera Phenix High Content Imaging System (Perkin Elmer). Briefly, cells grown in CellCarrier-96 Ultra Microplates were fixed with 4% Paraformaldehyde (Thermofisher) for 15 min at room temperature, washed in phosphate buffered saline, and treated with Hoechst 33342 (2 µg/ml; Sigma) to label nuclei. Wells were imaged in confocal mode. Digital-phase contrast images of the cell body were acquired alongside fluorescence images, and data were analysed using Harmony software. Cells were partitioned into nuclear and cytoplasmic subcellular regions. The intensity of TDP-43 within each subcellular region was quantified on a cell-by-cell basis, and average per-well data were used for downstream quantification.

### Primary Rat Cortical Neuron Cell Culture and Transfection

Cortices from embryonic day (E)19–20 Long-Evans rat embryos were dissected and disassociated, and primary neurons were plated at a density of 6 × 10^5^ cells/ml in 96-well plates, as described previously [35]. At in vitro day (DIV) 4, neurons were transfected with 100 ng EGFP to mark cell bodies and 50–100 ng of GFP-tagged TDP-43 constructs using Lipofectamine 2000 (Invitrogen 52887), as previously described [36–38]. Following transfection, cells were placed in Neurobasal Complete Media (Neurobasal (Gibco 21103-049), 1 × B27, 1 × Glutamax, 100 units/ml Pen Strep (Gibco 15140-122)) and incubated at 37 °C in 5% CO_2_. For compound treatments, neuronal media was supplemented at the time of transfection with either vehicle control or the GSK3 inhibitor CHIR99021 at concentrations ranging from 0.1 µM to 10 µM.

### Longitudinal Fluorescence Microscopy and Automated Image Analysis

Neurons were imaged as described previously [39, 40] using a Nikon Eclipse Ti inverted microscope with PerfectFocus3a 20X objective lens and an Andor Zyla4.2 (+) sCMOS camera. A Lambda XL Xenon lamp (Sutter) with a 5 mm liquid light guide (Sutter) was used to illuminate samples, and custom scripts written in Beanshell for use in µManager controlled all stage movements, shutters, and filters. Custom ImageJ/Fiji macros and Python scripts were used to identify neurons and draw both cellular and nuclear regions of interest (ROIs) based upon size, morphology, and fluorescence intensity. Fluorescence intensity of labelled proteins was used to determine protein localisation or abundance. Custom Python scripts were used to track ROIs over time, and cell death marked a set of criteria that include rounding of the soma, loss of fluorescence, and degeneration of neuritic processes [36]. For measurement of nuclear and cytoplasmic protein levels, we performed automated analysis as described [39, 41]. Briefly, Hoechst vital nuclear dye was applied immediately after transfection. Nuclear ROIs were established by automated segmentation of the DAPI channel, while cellular ROIs were identified via a similar process in the RFP channel (corresponding to mApple fluorescence). The intensity of TDP-43-GFP constructs was then measured within the nuclear and cellular ROIs of each neuron, and cytoplasmic levels were calculated as the difference between the cellular and nuclear ROIs.

#### Primary Motor Neuron Culture and Transfection

Primary motor neurons were isolated and cultured from embryonic day 13.5 mouse embryos as previously described [42, 43]. Briefly, lumbar spinal cords were dissected, digested with trypsin, and dissociated to a single-cell suspension. Primary motor neurons were isolated by density gradient centrifugation using 6% Optiprep (Sigma) and cultured on glass coverslips coated with 0.5 mg/ml poly-ornithine (Sigma) and 0.5 mg/ml laminin (Thermo Fisher Scientific). Neurons were maintained in Neurobasal/B27 medium supplemented with 2% horse serum (Sigma) and 10 ng/ml each of BDNF, CNTF, and GDNF (Peprotech) with 50% media exchanges every 3 days. Primary motor neurons were transfected by magnetofection as described [42]. Motor neurons were transfected at 2 DIV using magnetic nanobeads (NeuroMag, Oz Biosciences). Culture media was exchanged 1 h prior to transfection with Neurobasal/B27 medium without serum. Plasmid DNA was incubated with NeuroMag in minimal essential medium (MEM) for 15 min and then added dropwise to the cultures. Cells were incubated on top of a magnetic plate (Oz Biosciences) for 15 min, and after removal of the magnet, media were exchanged for complete neuronal media after 1 h.

### Motor Neuron Survival Assay

To quantify primary motor neuron survival, neurons were co-transfected with TDP-43 expression constructs and the pGL4.50[luc2/CMV/Hygro] luciferase reporter (Promega). After 4 DIV, luciferase expression was quantified using the Bio-Glo™ Luciferase Assay System (Promega) and a PHERAstar FS plate reader. Luciferase expression was used as a proxy for the number of surviving neurons. Assay reproducibility was confirmed by manual counting of GFP-TDP-43 positive cells.

#### Human Induced Pluripotent Stem Cell (iPSC)-Derived Forebrain Neuron Culture and Transfection

Human forebrain neurons were differentiated from a KOLF2.1J iPSC line with stable integration of a doxycycline-inducible neurogenin-2 (NGN2) expression cassette in the CLYBL safe harbour locus on chromosome 13. Stem cells were maintained in mTeSR plus media (Stemcell Technologies), routinely passaged using versene, and maintained on recombinant vitronectin-coated plates (Thermo Fisher Scientific). To differentiate neurons, stem cells were single-cell dissociated using accutase and replated onto Geltrex-coated dishes (Thermo Fisher Scientific) in stem cell media for 24 h with the addition of a ROCK inhibitor (Merck). On days 1, 2, and 3 post plating, media was exchanged for neuronal induction media consisting of DMEM-F-12/HEPES, 1 × N2, 1 × Glutamax, 1 × Non-essential amino acids (Thermo Fisher Scientific) with the addition of 2 mg/ml doxycycline (Merck) to induce expression of *NGN2*. After induction, neurons were dissociated with accutase treatment before replating into assay plates coated with a combination of Geltrex and laminin (Thermo Fisher Scientific). Neurons were maintained in neuronal maturation media consisting of Neurobasal Plus with the addition of 1× Glutamax, 10 ng/ml recombinant NT-3 (PeproTech), and 10 ng/ml recombinant BDNF (PeproTech) with media exchanged twice weekly (50%).

Neurons were transfected using magnetofection following the same protocol as for primary mouse motor neurons above, except that human forebrain-specific media was substituted for rodent neuron media.

#### Statistical Analyses

Statistical analyses were conducted using Prism 8.4.3 (GraphPad). For comparisons between genotypes or experimental groups, multiple *t*-tests or one-way ANOVA were used when comparing two or three groups, respectively. For comparison of means split on two independent variables, two-way ANOVA was used. Multiple comparisons were corrected using the Holm–Sidak test. For primary rat neuron survival analysis, the open-source R survival package was used to determine hazard ratios describing the relative survival between conditions through Cox proportional hazards analysis [36]. The statistical tests used and appropriate sample sizes are provided in the relevant figure legends. All statistical comparisons are based on biological replicates unless stated otherwise.

## Results

### TDP-43 Is Cleaved by Caspases at its N-terminus

To explore mechanistic links between TDP-43 and GSK3, we expressed wild-type (TDP-43^WT^) and ALS-linked mutant (TDP-43^Q331K^) isoforms in human SH-SY5Y neuroblastoma cells. To control for non-specific effects of overexpression, we also used an N-terminally truncated construct (TDP-43^N-Del^) lacking the first 81 amino acids (Δ1–81) (Fig. 1a). This deleted region encompasses the N-terminal domain required for dimerisation and self-oligomerisation, functions essential for nucleic acid binding [44], subcellular localisation, and aggregation propensity [45–48]. TDP-43^N-Del^ retains the nuclear localisation signal and a putative caspase cleavage site at Asp89. We posited that this construct would serve as a useful negative control. All isoforms were C-terminally GFP-tagged to facilitate detection and direct comparison (Fig. 1a).

Immunoblotting of GFP-tagged proteins revealed that cells expressing TDP-43^WT^ or TDP-43^Q331K^ exhibited two major bands: full-length TDP-43 and a smaller ~ 55 kDa species, comparable in size to GFP-tagged TDP-43^N-Del^ (Fig. 1b). As TDP-43^N-Del^ is truncated close to the Asp89 caspase recognition site, we hypothesised that the ~ 55 kDa band from the full-length isoforms represented a caspase cleavage product. Consistent with this, treatment with the pan-caspase inhibitor Q-VD-OPh significantly reduced the ~ 55 kDa fragment and increased the abundance of full-length TDP-43^WT^ and TDP-43^Q331K^ (Fig. 1b–e). Interestingly, TDP-43^N-Del^ levels also increased with Q-VD-OPh, suggesting that it was also cleaved at Asp89 (Fig. 1c). To verify the identity of GFP-positive bands, we re-probed with a pan–TDP-43 antibody, which detected the GFP-tagged species alongside endogenous full-length and low-abundance truncated TDP-43, confirming that the GFP-positive bands correspond to TDP-43 (Fig. 1b).

**Fig. 2:**
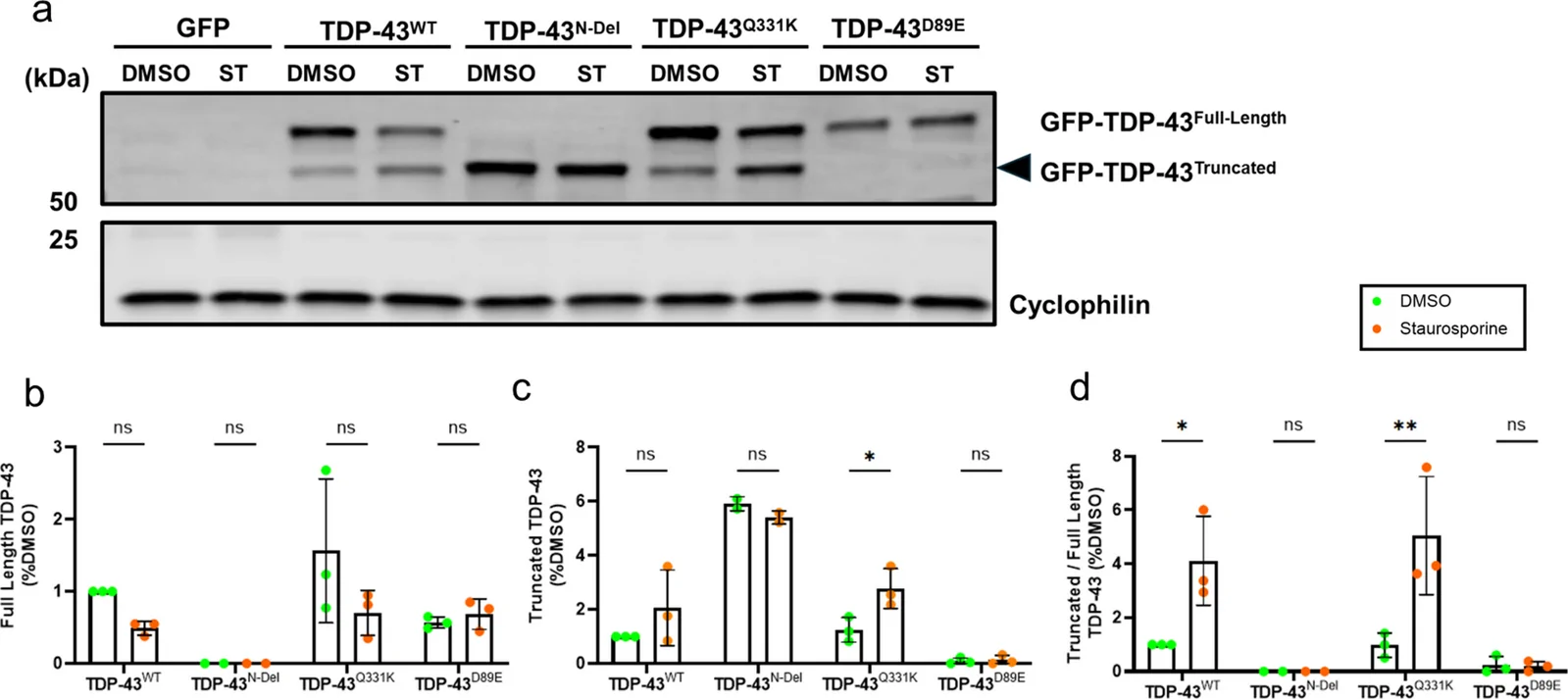
TDP-43 truncation is dependent on its N-terminal caspase recognition motif. **a** Representative immunoblot of SH-SY5Y cells transfected for 24 h with GFP alone or GFP-tagged TDP-43^N-Del^, TDP-43^WT^, TDP-43^Q331K^, or TDP-43^D89E^, with or without the potent pro-apoptotic caspase activator staurosporine. **b**–**d** Quantification of GFP-tagged TDP-43 immunoblot bands showing the abundance of full-length species (**b**), truncated species (**c**), and the truncated:full-length ratio (**d**) in the absence or presence of staurosporine (DMSO vs. staurosporine). Pairwise comparisons: full-length TDP-43 (**b**): ns (*P* = 0.4732) for TDP-43^WT^; ns (*P* > 0.9999) for TDP-43^N-Del^; ns (*P* = 0.0809); for TDP-43^Q331K^; ns (*P* = 0.9944) for TDP-43^D89E^. Truncated TDP-43 (c): ns (*P* = 0.1660) for TDP-43^WT^; ns (*P* = 0.6835) for TDP-43^N-Del^; *(*P* = 0.0413); for TDP-43^Q331K^; ns (*P* = 0.9450) for TDP-43^D89E^. Truncated:full-length ratio (**d**): ** (*P* = 0.0018) for TDP-43^WT^; ** (*P* = 0.0016) for TDP-43^Q331K^. For (**b**–**d**): *n* = 3 biological replicates per condition; two-way ANOVA followed by Holm–Sidak post-hoc tests for pairwise comparisons. Error bars denote mean ± s.e.m

To further confirm the relationship between caspase activity and TDP-43 processing, we treated cultures with staurosporine, a potent pro-apoptotic agent that activates caspases. Staurosporine markedly increased the ~ 55 kDa fragment in TDP-43^WT^ and TDP-43^Q331K^ (Fig. 2a–d) but had no effect on a cleavage-resistant TDP-43^D89E^ mutant, which lacks the Asp89 caspase recognition motif within the N-terminal nuclear localisation signal (NLS). Staurosporine did not alter the abundance of TDP-43^N-Del^, likely because any cleaved product would differ from the uncleaved protein by only a few amino acids, making them indistinguishable by SDS-PAGE. TDP-43^Q331K^ behaved similarly to TDP-43^WT^ under basal conditions and following either caspase inhibition or staurosporine-induced activation, indicating that the Q331K mutation may not exert toxicity through altered N-terminal caspase cleavage. Together, these results show that overexpressed TDP-43 undergoes caspase-dependent cleavage at Asp89 within the N-terminal NLS, generating C-terminal fragments.

### TDP-43 Activates GSK3 While TDP-43 Knockdown Inhibits GSK3

**Fig. 3:**
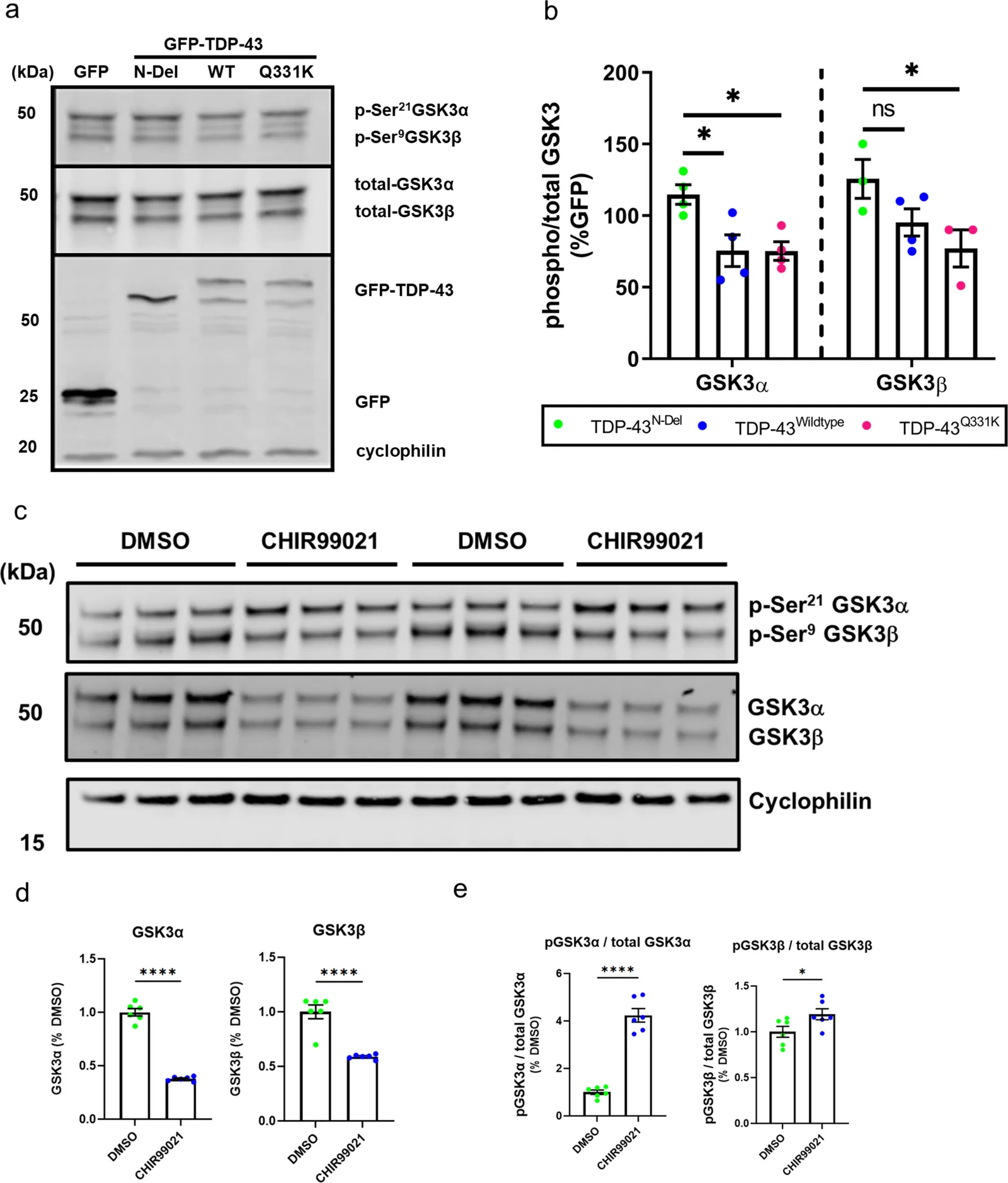
Selective activation of GSK3 by TDP-43 variants and inhibition by CHIR99021. **a** Representative immunoblot of SH-SY5Y neuroblastoma cells transfected for 24 h with GFP alone or GFP-tagged TDP-43 variants: N-terminal deletion (TDP-43^N-Del^; Δ1–81), wild type (TDP-43^WT^), or ALS-linked Q331K (TDP-43^Q331K^). Blots were probed for inhibitory phosphorylation of GSK3α (Ser21) and GSK3β (Ser9), and for total GSK3α/β. **b** Quantification of phospho-GSK3α or phospho-GSK3β normalised to total GSK3α or GSK3β, respectively, expressed relative to GFP control (*n* = 3–4 biological replicates per condition). A lower phospho/total ratio indicates greater GSK3 activity. Two-way ANOVA revealed a significant main effect of TDP-43 variant (*P* = 0.0012) with pairwise comparisons as follows: GSK3α: TDP-43^N-Del^ vs. TDP-43^WT^, * (*P* = 0.0186); TDP-43^N-Del^ vs. TDP-43^Q331K^, * (*P* = 0.0186). GSK3β: TDP-43^N-Del^ vs. TDP-43^WT^, ns (*P* = 0.0515); TDP-43^N-Del^ vs. TDP-43^Q331K^, * (*P* = 0.0123). **c** Representative immunoblot of SH-SY5Y cells treated for 24 h with vehicle (DMSO) or the selective GSK3 inhibitor CHIR99021 (10 µM), probed as in (**a**). **d** Quantification of total GSK3α and total GSK3β abundance (*n* = 6 per condition). **e** Quantification of phospho/total GSK3α and phospho/total GSK3β ratios. CHIR99021 significantly increased phospho-GSK3β (* *P* = 0.0473) and reduced total GSK3α/β abundance (**** *P* < 0.0001). For (a-b): n = 3–4 biological replicates per condition; two-way ANOVA followed by Holm–Sidak post-hoc tests for pairwise comparisons. For (c-e): n = 6 biological replicates per condition; one-way ANOVA followed by Holm–Sidak post-hoc tests for pairwise comparisons. Error bars denote mean ± s.e.m

Immunoblotting of cell lysates for GSK3 demonstrated that expression of TDP-43^Q331K^ significantly reduced inhibitory phosphorylation of both GSK3α (Ser21) and GSK3β (Ser9), consistent with activation of both isoforms [49–51]. TDP-43^WT^ also significantly reduced phosphorylation of GSK3α, while showing a non-significant trend toward reduced GSK3β phosphorylation. In contrast, TDP-43^N-Del^ had no significant effect on phosphorylation of either isoform, suggesting that N-terminal dimerisation is required for full activation of GSK3 by TDP-43 (Fig. 3a–b). These observations align with prior studies showing that TDP-43 can activate GSK3 in cellular models [31].

To further examine the role of TDP-43 in regulating endogenous GSK3 activity, we performed reciprocal siRNA-mediated knockdown of *TARDBP* in SH-SY5Y neuroblastoma cells, achieving a mean knockdown of 75% TDP-43 protein by 72 h (Supplementary Fig. 1a–c). Interestingly, depletion of TDP-43 led to a significant increase in inhibitory phosphorylation of both GSK3α and GSK3β (Supplementary Fig. 1b, d). In parallel, total levels of both GSK3α and GSK3β were significantly reduced (Supplementary Fig. 1b, e). This suggests that TDP-43 dynamically modulates GSK3 function by influencing both its abundance and its activity.

### GSK3 Inhibitor CHIR99021 Reduces GSK3 Abundance and Increases GSK3 Inhibitory Phosphorylation

To further explore the role of GSK3 in our neuronal studies, we opted to use the GSK3 inhibitor CHIR99021, a widely used, potent, and specific GSK3 inhibitor [52]. CHIR99021 is thought to compete for binding at the ATP binding site of GSK3. Surprisingly, we found that treatment of SH-SY5Y cells with CHIR99021 for 24 h resulted in a significant increase in the phosphorylation of both GSK3α and GSK3β, as well as a significant decrease in the abundance of total GSK3α and GSK3β (Fig. 3c–e).

To determine whether CHIR99021 exerts similar effects in human neurons, we generated human forebrain neurons from induced pluripotent stem cells (iPSCs) via doxycycline-inducible expression of neurogenin-2 from a stably integrated transgene (Supplementary Fig. 2a). Neuronal differentiation was confirmed by immunofluorescence for microtubule-associated protein 2 (MAP2) and neuronal nuclear antigen (NeuN), with a mean purity of 92% achieved by day 14 of culture (Supplementary Fig. 2b–c). CHIR99021 similarly reduced the abundance of both GSK3α and GSK3β in these iPSC-derived forebrain neurons, while preferentially increasing GSK3α inhibitory phosphorylation (Supplementary Fig. 3a–c). To investigate the mechanism underlying reduced GSK3 protein levels, we performed RT-qPCR in SH-SY5Y cells treated with CHIR99021 and observed a significant reduction in *GSK3A* and *GSK3B* mRNA expression, suggesting that transcriptional downregulation contributes to the observed effects (Supplementary Fig.  3 d). Collectively, these results suggest that CHIR99021 can inhibit GSK3 function by reducing its transcription and by increasing post-translational inhibitory phosphorylation.

### GSK3 Inhibition Preferentially Reduces the Abundance of Truncated TDP-43

**Fig. 4:**
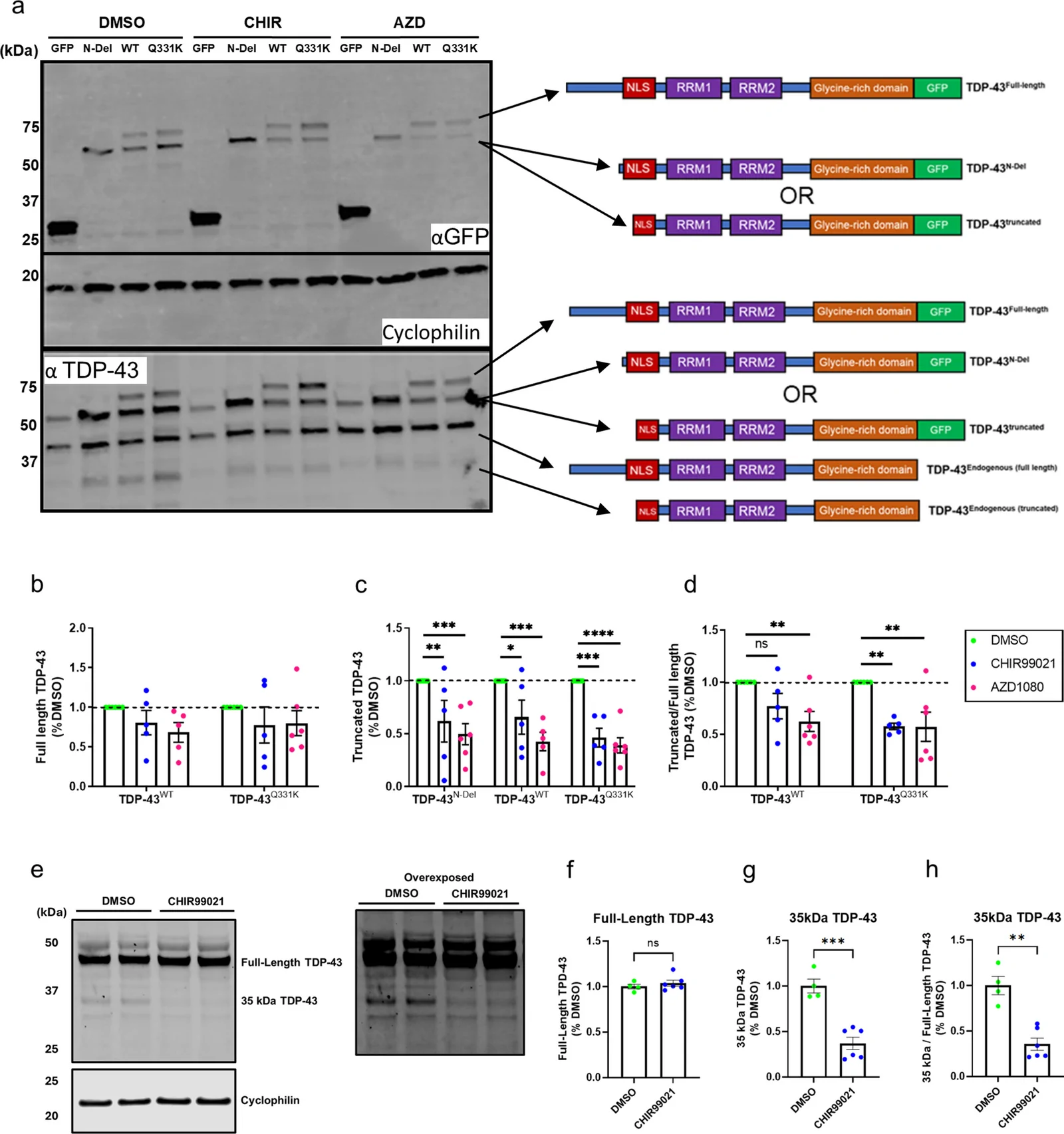
GSK3 inhibition preferentially reduces the abundance of truncated TDP-43. **a** Representative immunoblot of SH-SY5Y cells transfected for 24 h with GFP alone or GFP-tagged TDP-43^N-Del^, TDP-43^WT^, or TDP-43^Q331K^, and treated with the GSK3 inhibitors CHIR99021 (CHIR) or AZD1080 (AZD). **b**–**d** Quantification of GFP-tagged TDP-43 immunoblot bands showing the abundance of full-length species (**b**), truncated species (**c**), and the truncated:full-length ratio (**d**) following treatment with CHIR or AZD. **b** Full-length TDP-43: ANOVA treatments, ns (*P* = 0.1216). **c** Truncated TDP-43, pairwise comparisons: TDP-43^N-Del^, CHIR vs DMSO, **(*P* = 0.0077); AZD vs DMSO, ***(*P* = 0.0007); TDP-43^WT^, CHIR vs DMSO, *(*P* = 0.0156); AZD vs DMSO, ***(*P* = 0.0003); TDP-43^Q331K^, CHIR vs DMSO, ***(*P* = 0.0003); AZD vs DMSO, ****P < 0.0001. **d** Ratio truncated:full-length TDP-43, pairwise comparisons: TDP-43^WT^, CHIR vs DMSO, ns (*P* = 0.0752); AZD vs DMSO, **(*P* = 0.0071); TDP-43^Q331K^, CHIR vs DMSO, **(*P* = 0.0024); AZD vs DMSO, **(*P* = 0.0024). **e** Representative immunoblot of SH-SY5Y cells treated with CHIR99021 (24 h) and probed for endogenous TDP-43, with an overexposed panel to visualise the low-abundance 35 kDa fragment. **f**–**h** Quantification of endogenous TDP-43 showing full-length TDP-43 (**f**) and the 35 kDa fragment (**g**, **h**) following CHIR treatment. **f** Full-length TDP-43: ns (*P* = 0.3930); (**g**) truncated TDP-43 relative to cyclophilin loading control ***(*P* = 0.0005); (h) truncated TDP-43 relative to full-length TDP-43 **(*P* = 0.0021). For (b–d): n = 5–6 biological replicates per condition; two-way ANOVA followed by Holm–Sidak post-hoc test for pairwise comparisons. For (f–h): n = 4–6 biological replicates per condition; Unpaired t-test with Welch’s correction. Error bars denote mean ± s.e.m

To assess whether GSK3 inhibition influences TDP-43 fragmentation, SH-SY5Y neuroblastoma cells expressing TDP-43^N-Del^, TDP-43^WT^, or TDP-43^Q331K^ were treated with CHIR99021 (Fig. 4a). We also used a structurally and mechanistically distinct GSK3 inhibitor, AZD1080, to control for compound-specific artefacts. While changes in full-length TDP-43^WT^ and TDP-43^Q331K^ abundance were modest (Fig. 4b), both GSK3 inhibitors produced a significant reduction in the cleaved products of TDP-43^WT^ and TDP-43^Q331K^ (Fig. 4c, d). In addition, the abundance of TDP-43^N-Del^ was also reduced (Fig. 4c), indicating that this truncated construct remains subject to regulation by GSK3-dependent pathways.

To determine whether GSK3 inhibition also impacts endogenous TDP-43 processing, we probed SH-SY5Y lysates with an antibody sensitive to the detection of truncated endogenous TDP-43 (Abcam ab57105) (Fig. 4e). Treatment with CHIR99021 for 24 h did not alter the abundance of full-length endogenous TDP-43 (Fig. 4e, f) but significantly reduced the 35 kDa cleaved fragment by > 50%, whether quantified relative to loading control (cyclophilin; Fig. 4g) or to full-length TDP-43 (Fig. 4h). In addition, we probed lysates from CHIR99021-treated SH-SY5Y neuroblastoma cells (Supplementary Fig. 4a–b) and iPSC-derived forebrain neurons (Supplementary Fig. 4c–d) with a second commercial TDP-43 antibody (Proteintech 10782-2-AP). In both cell types, this antibody corroborated the lack of change in full-length TDP-43 but did not reliably detect the 35 kDa fragment, underscoring key differences in antibody-dependent sensitivity for the detection of cleaved products.

To test the converse, whether GSK3 influences endogenous TDP-43 cleavage, we overexpressed GSK3α, GSK3β, or a combination of both isoforms in SH-SY5Y cells and probed for endogenous TDP-43 (Supplementary Fig. 5a). Immunoblotting confirmed robust overexpression of both kinases, with GSK3α generally achieving higher steady-state levels than GSK3β under identical transfection conditions. GSK3β migrated at a slightly higher apparent molecular weight, consistent with the myc-tagged construct we used and/or isoform differences in SH-SY5Y cells. Full-length endogenous TDP-43 was unchanged across conditions (Supplementary Fig. 5b). In contrast, the 35 kDa cleaved TDP-43 fragment increased upon overexpression of either GSK3α or GSK3β. This effect was evident as a trend when normalised to the loading control (cyclophilin; Supplementary Fig. 5c) and statistically significant when measured relative to full-length TDP-43 levels (Supplementary Fig.  5d). Therefore, elevating GSK3 activity increases the abundance of endogenous cleaved TDP-43.

Collectively, our results show that GSK3 inhibition and activation influence cleavage of endogenous TDP-43 in opposite directions without significant changes in full-length TDP-43.

### GSK3 Inhibition Reduces the Level of Nuclear TDP-43 in a Caspase-Dependent Manner

**Fig. 5:**
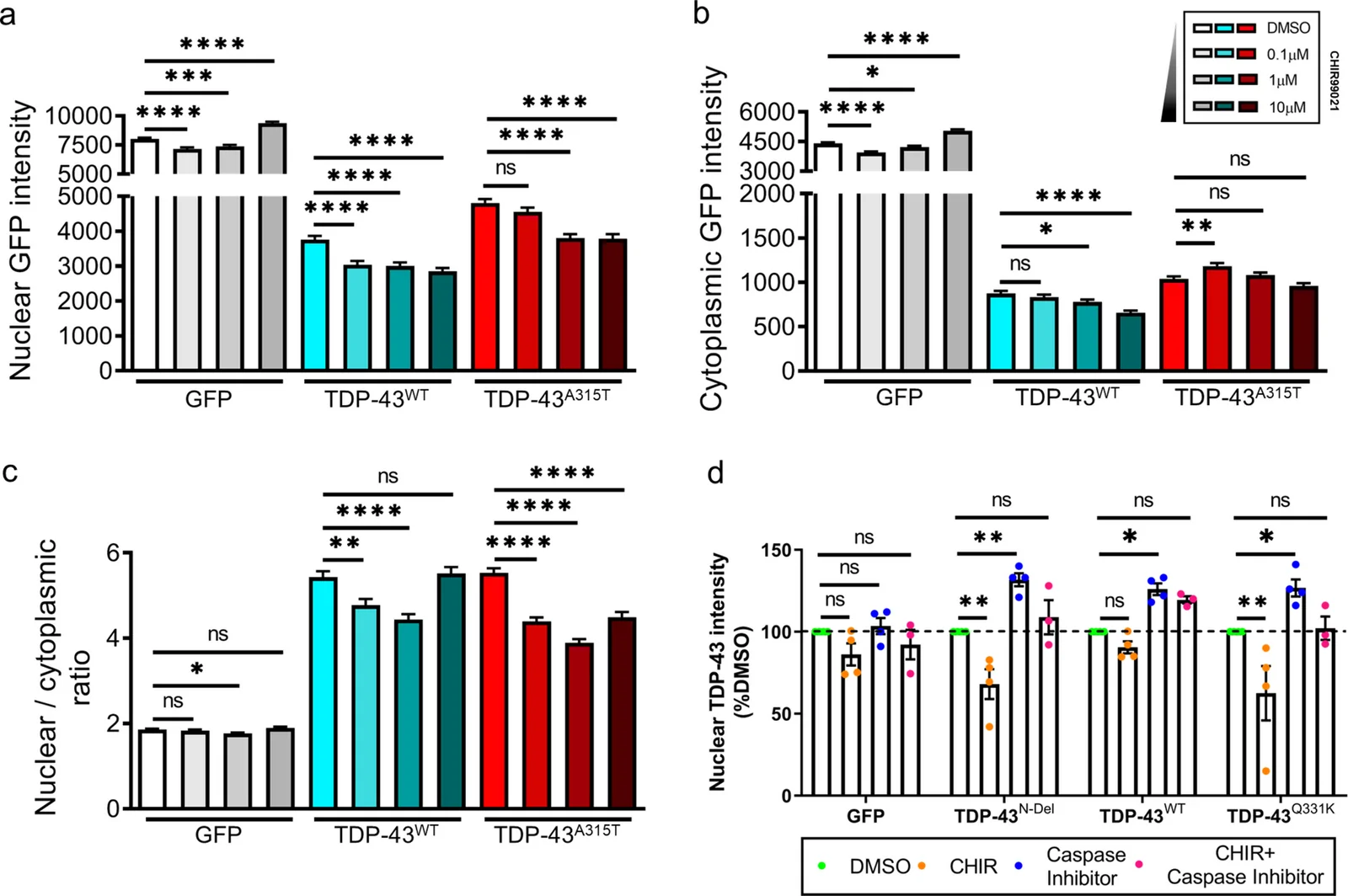
Inhibition of GSK3 reduces nuclear TDP-43 abundance through a caspase-dependant mechanism. **a**–**c** Subcellular distribution and abundance of GFP or GFP-tagged TDP-43^WT^ and TDP-43^A315T^ in primary rat cortical neurons treated with increasing doses of the GSK3 inhibitor CHIR99021. **a** Nuclear TDP-43, pairwise comparisons: GFP, 1 µM CHIR, ***(*P* = 0.0003); TDP-43^A315T^, 0.1 µM CHIR, ns (*P* = 0.1132). **b** Cytoplasmic TDP-43, pairwise comparisons: GFP, 1 µM CHIR, *(*P* = 0.0256); TDP-43^WT^, 0.1 µM CHIR, ns (*P* = 0.2529), 1 µM CHIR, *(*P* = 0.0292); TDP-43^A315T^, 0.1 µM CHIR, **(*P* = 0.0023), 1 µM CHIR, ns (*P* = 0.3181), 10 µM CHIR, ns (*P* = 0.1379). **c** Ratio nuclear:cytoplasmic TDP-43, pairwise comparisons: GFP, 0.1 µM CHIR, ns (*P* = 0.4525), 1 µM CHIR, *(*P* = 0.0110), 10 µM CHIR, ns (*P* = 0.3138); TDP-43^WT^, 0.1 µM CHIR, **(*P* = 0.0018), 10 µM CHIR, ns (*P* = 0.6625). For (a–c): *n* = 619–991 cells per condition from 3 technical replicate experiments; ****P < 0.0001; one-way ANOVA with Holm–Sidak post-hoc test. (**d**) Nuclear abundance of GFP-tagged TDP-43^N-Del^, TDP-43^WT^, or TDP-43^Q331K^ in SH-SY5Y cells treated for 24 h with CHIR99021, the pan-caspase inhibitor Q-VD-OPh, or both in combination. Pairwise comparisons: GFP, CHIR, ns (*P* = 0.4423); caspase inhibitor, ns (*P* = 0.9809); CHIR + caspase inhibitor, ns (*P* = 0.8586). TDP-43^N-Del^, CHIR, **(*P* = 0.0062); caspase inhibitor, **(*P* = 0.0065); CHIR + caspase inhibitor, ns (*P* = 0.8069). TDP-43^WT^, CHIR, ns (*P* = 0.7312); caspase inhibitor, *(*P* = 0.0341); CHIR + caspase inhibitor, ns (*P* = 0.2179). TDP-43^Q331K^, CHIR, **(*P* = 0.0010); caspase inhibitor, *(*P* = 0.0278); CHIR + caspase inhibitor, ns (*P* = 0.9962). For (d): *n* = 3–4 biological replicates per condition; two-way ANOVA with Holm–Sidak post-hoc test. Error bars denote mean ± s.e.m

Cytoplasmic mislocalisation and nuclear depletion of TDP-43 are hallmarks of TDP-43 proteinopathies, particularly at end-stage disease. To investigate the effects of GSK3 inhibition on TDP-43 localisation, primary rat cortical neurons were transfected with TDP-43 constructs C-terminally tagged with GFP (either TDP-43^WT^ or ALS-linked mutant TDP-43^A315T^). Neurons were subsequently treated with CHIR99021 in doses ranging from 0.1 µM to 10 µM. TDP-43-GFP intensity in the cytoplasm and nucleus was determined by automated high-content fluorescence microscopy, using a vital nuclear dye (Hoechst) as reference for the nuclear compartment, and a diffusely localised cellular marker (mApple) to outline the neuronal cytoplasm [39]. GSK3 inhibition by CHIR99021 significantly reduced the nuclear abundance of both TDP-43^WT^ and TDP-43^A315T^ in a dose-dependent manner (Fig. 5a). The effect on cytoplasmic TDP-43 was less pronounced, and was influenced by TDP-43 genotype: higher doses of the GSK3 inhibitor significantly reduced the abundance of TDP-43^WT^ but not TDP-43^A315T^ (Fig. 5b), thereby causing a reduction in the nuclear to cytoplasmic ratio of both TDP-43^WT^ and TDP-43^A315T^ (Fig. 5c). Given that the vast majority of TDP-43 is localised to the nucleus (Fig. 5a, b), inhibition of GSK3 effectively reduces the abundance of total cellular TDP-43^WT^ and TDP-43^A315T^ in a dose-dependent manner.

Given that both caspase inhibition and GSK3 inhibition reduce N-terminally truncated TDP-43 (Fig. 1b, c; Fig. 4a, c), we hypothesised that caspase-mediated cleavage, by disrupting the NLS, is a key step in GSK3-dependent regulation of nuclear TDP-43. To test this, we utilised an additional experimental system, overexpressing GFP-tagged TDP-43^N-Del^, TDP-43^WT^, or TDP-43^Q331K^ in SH-SY5Y cells and treating them with either CHIR99021, the pan-caspase inhibitor Q-VD-OPh, or both in combination. Inhibition of GSK3 alone reduced nuclear TDP-43^N-Del^ and TDP-43^Q331K^, whereas caspase inhibition alone had the opposite effect, increasing nuclear abundance of all three variants (Fig. 5d). Strikingly, caspase inhibition blocked the ability of CHIR99021 to reduce nuclear TDP-43. This suggests that GSK3 regulates the abundance of TDP-43 through an N-terminal caspase cleavage-dependent mechanism. Notably, TDP-43^N-Del^, despite lacking the N-terminal dimerisation domain and not activating GSK3, was reduced in the nucleus upon CHIR99021 treatment. The retention of the NLS and Asp89 caspase site in TDP-43^N-Del^ likely explains why it is regulated in a similar way to full-length TDP-43^WT^ and TDP-43^Q331K^.

### GSK3 Inhibition Ameliorates TDP-43 Toxicity

**Fig. 6:**
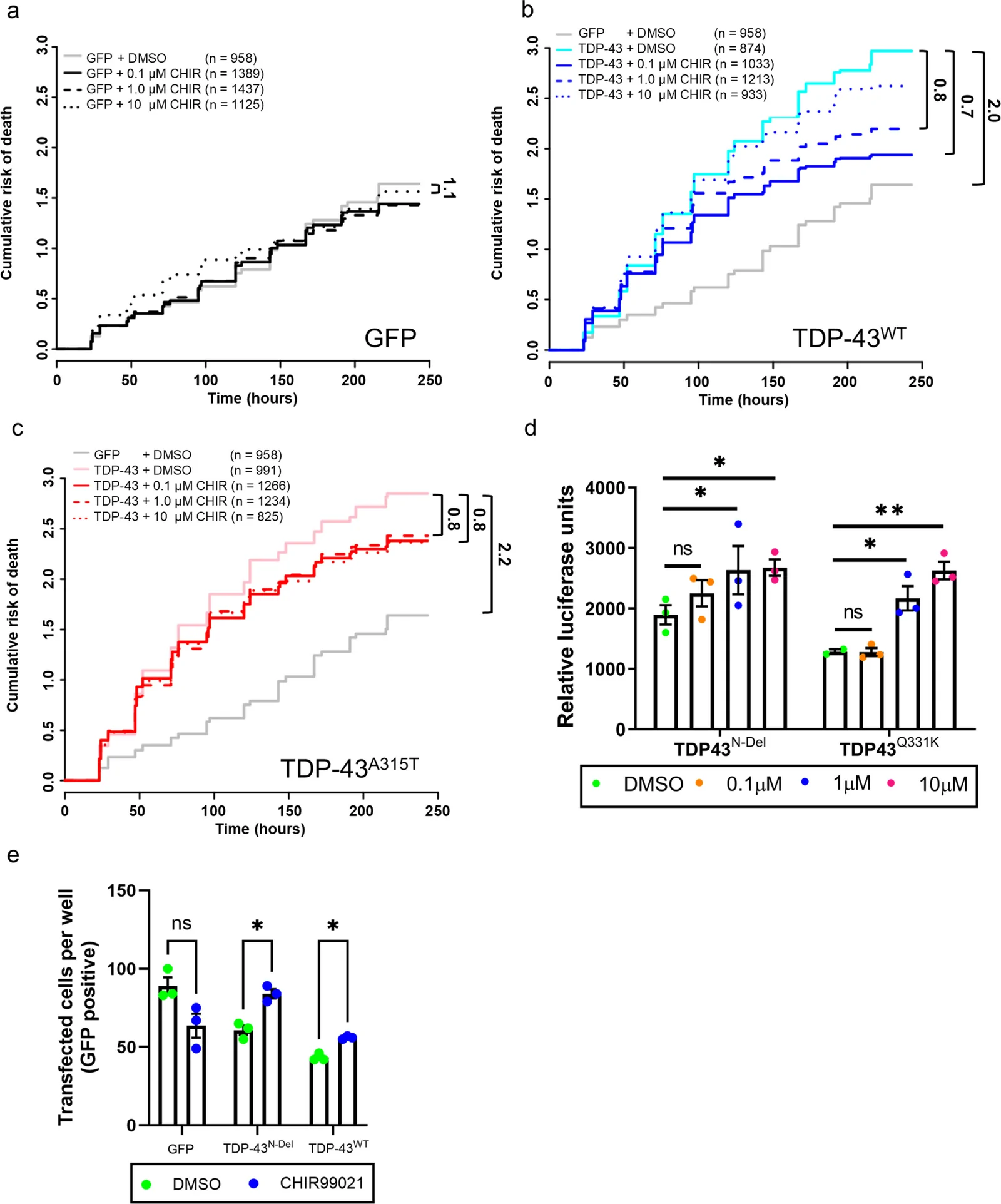
Small molecule inhibition of GSK3 ameliorates TDP-43 toxicity in rodent neurons and human iPSC-derived neurons. **a**–**c** Cumulative risk of death for primary rat cortical neurons expressing GFP or GFP-tagged TDP-43^WT^ or TDP-43^A315T^, treated with DMSO or increasing doses of CHIR99021, assessed by longitudinal fluorescence microscopy. A hazard ratio (HR) > 1.0 indicates increased risk of death; HR < 1.0 indicates reduced risk. **a** GFP-expressing neurons: GFP vs GFP + 0.1 µM CHIR, HR = 1.1 (significant). **b** TDP-43^WT^-expressing neurons: GFP vs TDP-43^WT^, HR = 2.0; TDP-43^WT^ vs TDP-43^WT^ + 0.1 µM CHIR, HR = 0.7; TDP-43^WT^ vs TDP-43^WT^ + 1.0 µM CHIR, HR = 0.8. **c** TDP-43^A315T^-expressing neurons: GFP vs TDP-43^A315T^, HR = 2.2; TDP-43^A315T^ vs TDP-43^A315T^ + 0.1 µM CHIR, HR = 0.8; TDP-43^A315T^ vs TDP-43^A315T^ + 1.0 µM CHIR, HR = 0.8. The number of individual neurons tracked for each risk-of-death analysis is shown. **d** Survival of primary mouse motor neurons expressing TDP-43^N-Del^ or TDP-43^Q331K^ after treatment with CHIR. Pairwise comparisons: TDP-43^N-Del^, 0.1 µM CHIR, ns (*P* = 0.2381); 1.0 µM CHIR, *(*P* = 0.0492); 10 µM CHIR, *(*P* = 0.0492). TDP-43^Q331K^, 0.1 µM CHIR, ns (*P* = 0.9825); 1.0 µM CHIR, *(*P* = 0.0312); 10 µM CHIR, **(*P* = 0.0026). For (d): n = 3 biological replicates per condition; two-way ANOVA with Holm–Sidak post-hoc test. **e** Survival of iPSC-derived forebrain neurons expressing GFP, TDP-43^N-Del^, or TDP-43^WT^ after CHIR treatment. Pairwise comparisons (DMSO vs CHIR99021): GFP, ns (*P* = 0.2381); TDP-43^N-Del^, ns (*P* = 0.2381); TDP-43^WT^, ns (*P* = 0.2381). For (e): n = 3 biological replicates per condition; multiple t-test with Holm–Sidak correction. Error bars denote mean ± s.e.m

We previously found that loss of GSK3 suppressed TDP-43-mediated neurodegeneration in *Drosophila melanogaster* [34]. To assess the therapeutic potential of GSK3 inhibition in mammalian systems, we first expressed GFP-tagged TDP-43^WT^ or ALS-linked TDP-43^A315T^ in primary rat cortical neurons and treated them with CHIR99021. Neuronal viability was tracked longitudinally using robotic microscopy [36]. Expression of either TDP-43^WT^ or TDP-43^A315T^ significantly increased the cumulative risk of death compared to GFP alone, with hazard ratios of 2.0 and 2.2 respectively (Fig. 6a–-c). CHIR99021 treatment reduced the risk of death in TDP-43-expressing neurons at specific concentrations.

We next tested whether this protective effect extended to primary mouse motor neurons, using a luciferase-based survival assay. Consistent with our rat cortical neuron findings, CHIR99021 significantly improved survival in motor neurons expressing TDP-43^Q331K^ (Fig. 6d). Interestingly, TDP-43^N-Del^ also demonstrated modest toxicity that was partially mitigated by GSK3 inhibition. We speculate that while TDP-43^N-Del^ does not have full functional capacity due to impaired ability to dimerise, it may exert toxicity because it retains the C-terminal glycine-rich low complexity domain, which allows it to interact with other proteins.

Finally, we examined if GSK3 inhibition modifies TDP-43 toxicity in human iPSC-derived forebrain neurons. CHIR99021 significantly increased survival in neurons expressing either TDP-43^WT^ or TDP-43^N-Del^ (Fig. 6e). Together, these results show that the survival-promoting effects of GSK3 inhibition are conserved across species (rat, mouse, human) and across neuronal subtypes (cortical, motor), indicating broad neuroprotective potential for GSK3 inhibition in TDP-43 proteinopathies.

## Discussion

### GSK3 Activity Regulates TDP-43 Proteostasis via a Caspase-Dependent Mechanism

**Fig. 7:**
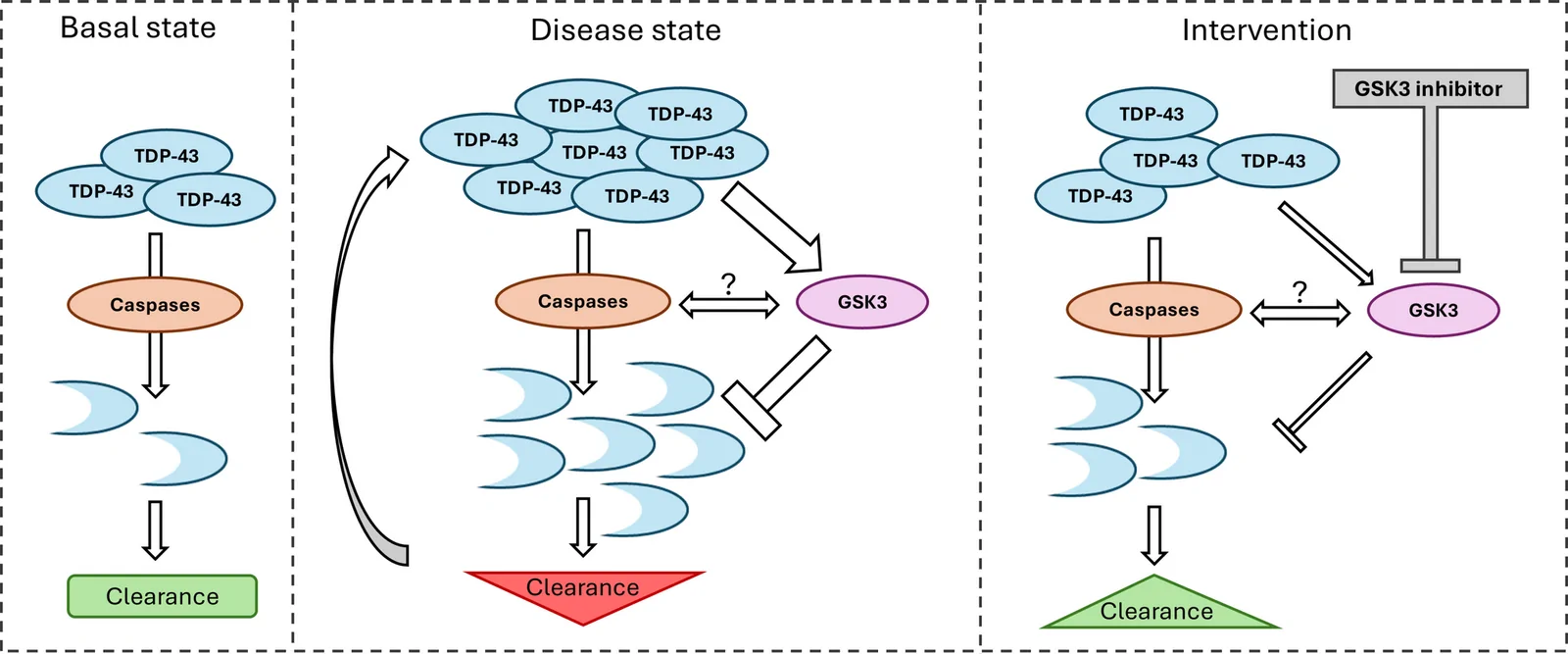
A proposed model linking GSK3 activity to TDP-43 proteostasis. Schematic representation of three states in the regulation of TDP-43 turnover and toxicity. (1) Basal state: Under physiological conditions, TDP-43 undergoes limited caspase-mediated cleavage. C-terminal fragments are efficiently cleared by proteasomal and autophagic pathways, maintaining TDP-43 homeostasis. (2) Disease state: Increased TDP-43 expression, altered GSK3 activity, increased caspase processing, or reduced TDP-43 clearance can all lead to a breakdown in TDP-43 proteostasis. TDP-43 can activate GSK3, which may then promote caspase-mediated TDP-43 cleavage. GSK3 activation inhibits clearance of TDP-43 fragments, which accumulate, resulting in protein turnover mechanisms becoming saturated. This may then lead to a build-up of TDP-43, which in turn promotes GSK activation, resulting in a positive feedback loop that perpetuates TDP-43 fragment build-up and neurotoxicity. (3) Intervention state: Inhibition of GSK3 interrupts this pathogenic cycle by reducing GSK3 activity, lowering the abundance of cleaved and full-length TDP-43, and improving neuronal survival. This model would suggest that GSK3 is a mechanistic regulator of TDP-43 proteostasis and a potential therapeutic target in TDP-43 proteinopathies

Our data support a complex mechanistic model in which GSK3 activity governs TDP-43 turnover and toxicity via caspase-mediated cleavage (Fig. 7). Under basal conditions, physiological levels of TDP-43 undergo limited caspase cleavage as part of homeostatic turnover, with C-terminal fragments subsequently cleared by proteasomal or autophagic mechanisms. However, in disease-relevant contexts, whether due to increased TDP-43 levels, impaired clearance, aberrant caspase activity, or increased GSK3 activation, this balance may be disrupted, promoting the accumulation of TDP-43 fragments, overwhelming proteostatic mechanisms and driving pathology. We did not directly quantify caspase enzymatic activity in our assays but infer caspase involvement from pharmacological manipulation (Q-VD-OPh versus staurosporine) and genetic resistance of the D89E mutant to truncation. These complementary approaches support a caspase-dependent step in TDP-43 processing in our models.

We demonstrate that overexpression of wild-type or mutant TDP-43 activates GSK3, enhances caspase cleavage, and leads to increased levels of truncated TDP-43 species. This cascade likely reflects a broader vulnerability in TDP-43 proteinopathies, wherein perturbations at multiple levels can converge to disrupt proteostasis. Pharmacological inhibition of GSK3 dampens this cycle, lowering both full-length and cleaved TDP-43, and significantly improving neuronal survival across rodent and human iPSC-derived models. We propose that GSK3 modulates the accumulation and turnover of TDP-43 species downstream of cleavage, rather than directly activating caspases. Although we did not directly measure protein clearance, the reduction in TDP-43 abundance and reduced neurotoxicity upon GSK3 inhibition suggests there has been a restoration of homeostatic turnover of TDP-43.

Our findings also reveal that caspase-mediated processing can act independently of GSK3 activation. The N-terminally truncated variant TDP-43^N-Del^, which lacks the dimerisation domain and does not activate GSK3, shows partial caspase susceptibility and modest toxicity. Notably, its abundance decreases with GSK3 inhibition, consistent with GSK3 controlling the steady-state levels of TDP-43 cleavage products and/or full-length protein without necessarily altering the initiation of caspase cleavage.

Our work also implicates caspases in TDP-43 nuclear localisation. Caspase inhibition increased nuclear TDP-43 levels even in the absence of GSK3 inhibition, indicating that caspase activity contributes to the regulation of TDP-43 localisation through both GSK3-dependent and GSK3-independent mechanisms. This complexity is further highlighted by the behaviour of TDP-43^N-Del^, which, although deficient in RNA binding and self-oligomerisation capacity, may still engage with other proteins, potentially influencing its subcellular distribution, stability, and susceptibility to cleavage.

In parallel, we show that CHIR99021 increases inhibitory phosphorylation (Ser21/9) and reduces total protein and transcript levels of GSK3α and GSK3β. These appear to be novel mechanisms by which CHIR99021 functions to inhibit GSK3. The two GSK3 isoforms show differential phosphorylation patterns, possibly due to distinct regulation, localisation, or substrate specificity. Further investigation is warranted to dissect isoform-specific contributions to TDP-43 processing and neuronal viability.

Together, our work uncovers a complex system in which TDP-43 is processed by caspases and activates GSK3 to regulate fragment accumulation and abundance. In turn, GSK3 inhibition restores proteostasis and protects neurons. This solidifies GSK3 as a mechanistic regulator and potentially a therapeutic target in TDP-43-linked neurodegenerative diseases.

### A Positive Feedback Loop in the TDP-43–GSK3 Axis Could Contribute to Neurodegeneration

In support of our findings, a growing body of evidence indicates that inhibition of GSK3 is neuroprotective. GSK3 inhibition significantly delays disease onset and prolongs lifespan in the SOD1^G93A^ mouse model of ALS [53–55], and the GSK3 inhibitor kenpaullone prolongs survival of human iPSC-derived motor neurons harbouring the SOD1^L144F^ or TDP-43^M337V^ mutations [56]. Chronic inhibition of GSK3 by lithium is neuroprotective against kainate-induced excitotoxic motor neuron death in organotypic slice cultures [57]. Ghrelin, a circulating hormone produced by enteroendocrine cells, protects spinal motor neurons against glutamate-induced excitotoxicity in part through PI3K/Akt-mediated inactivation of GSK3β [58]. Inhibitors of GSK3 abrogate accumulation of C-terminal TDP-43 fragments in transfected cells [33] and protect motor neurons from neuroinflammation-induced degeneration [59]. Thus, GSK3 is an attractive target for therapeutic intervention in TDP-43-linked neurodegeneration.

While inhibition of GSK3 influences TDP-43 abundance, it is also notable that TDP-43 can activate GSK3 [32]. Furthermore, the abundance of GSK3β is increased in the frontal and temporal cortices of patients with ALS and concomitant cognitive impairment [30]. Expression of TDP-43 perturbs the ER–mitochondria interface by disrupting interaction between VAPB and PTPIP51 through GSK3β activation [32]. Thus, TDP-43 and GSK3 are fundamentally linked in a reciprocal manner, with misregulation of one impacting the function of the other. This intimate relationship raises the possibility that elevated TDP-43 could act in a positive feedback loop by activating GSK3 to negatively impact its own turnover. In such a situation, the abundance of TDP-43 would increase over time as its GSK3-mediated clearance is progressively impaired (Fig. 7).

Although TDP-43 overexpression activates GSK3 and TDP-43 is processed by caspases, it remains unclear whether these pathways act independently or intersect to modulate TDP-43 proteolysis and toxicity. Our current data do not directly address this interplay, and future work will be required to determine whether GSK3 inhibition modulates caspase activation.

### TDP-43 Abundance Must Be Tightly Controlled for Cellular Viability

TDP-43 binds a large proportion of the transcriptome and regulates multiple steps of RNA metabolism [2, 60–64]. Even minor alterations in TDP-43 abundance can cause widespread transcriptomic changes that compromise cellular function, underscoring the need for precise homeostatic control [65, 66]. This control is achieved in part through an autoregulatory feedback mechanism, disruption of which is associated with ALS-FTD [65, 67–70]. Our results suggest that dysregulation of the caspase-dependent GSK3–TDP-43 axis represents another pathway through which TDP-43 levels may become pathologically altered.

At the endoplasmic reticulum (ER), TDP-43 can be cleaved at amino acid 174 by membrane-bound caspase-4 to generate a 25 kDa C-terminal fragment [24, 71]. Subsequent activation of caspase-3/7 produces a 35 kDa fragment. This sequential processing reduces the abundance of full-length TDP-43 and mitigates cytotoxicity caused by its overexpression [24]. Notably, TDP-43 overexpression initiates caspase-4 cleavage before the onset of detectable ER stress, suggesting that cleavage at the ER represents a physiological regulatory mechanism rather than simply a response to stressors [25]. Thus, both RNA-level and protein-level mechanisms act in concert to keep TDP-43 abundance within a narrow range critical for cell viability.

### Misregulation of TDP-43 in Neurodegenerative Disease

The accumulation and aggregation of TDP-43 in ALS-FTD brain tissue suggests that these homeostatic mechanisms become overwhelmed during disease [3]. Disruption can arise through multiple routes. For example, the ALS-associated Q331K mutation perturbs autoregulation and increases TDP-43 abundance [65]. Patient-derived TDP-43^M337V^ neurons show elevated TDP-43 expression [72], and spinal motor neurons from patients with apparently sporadic ALS exhibit increased TARDBP mRNA [73]. Regulatory elements in the untranslated regions (UTRs) of *TARDBP* influence transcript stability and turnover, and rare variants in these regions are enriched in ALS [74]. One such variant (c.*2076G > A in two ALS-FTD patients) was shown to double *TARDBP* mRNA levels [75]. Under these conditions, the proteolytic processing of TDP-43, such as that occurring at the ER, could be saturated, contributing to TDP-43 misregulation.

### TDP-43 Fragmentation Is Increased in Disease

Caspase activation occurs in several ER stress contexts [76] including ageing [77], protein misfolding [78] and aggregation [79], and is increased in the brains and spinal cords of ALS patients [80]. Various insults, including chemically induced apoptosis, ER stress, chronic oxidative stress, and D-sorbitol-induced hyperosmotic pressure, trigger caspase-dependent generation of 35 kDa TDP-43 fragments [25].

Mutant TDP-43 can be intrinsically more prone to fragmentation: human lymphoblastoid lines from *TARDBP* mutation carriers exhibit enhanced cleavage [5, 15, 81], and overexpression of TDP-43^A315T^ in HEK293 cells leads to persistent accumulation of protease-resistant fragments [82]. In C9orf72-associated ALS-FTD, poly-GA dipeptide repeats formed by repeat-associated non-AUG (RAN) translation of the hexanucleotide repeat expansion induce caspase-3 expression [83]. This could potentially lead to increased TDP-43 proteolysis. Activated caspase-3 levels are also elevated in spinal motor neurons of ALS patients carrying risk-modifying polyglutamine expansions in ATXN2 [84].

Although C-terminal TDP-43 fragments are a pathological hallmark of ALS-FTD, overexpression of 35 kDa or 25 kDa fragments does not always cause neurodegeneration in vivo or cell death in vitro [85, 86]. This supports the view that caspase-mediated cleavage can, under some conditions, attenuate toxicity. Further work will be needed to determine when TDP-43 fragmentation is protective versus pathogenic, and whether this mechanism can be therapeutically leveraged to reduce TDP-43-linked neurodegeneration.

### Study Limitations

Our study employs a transient overexpression model of TDP-43, which does not fully recapitulate the physiological regulation of endogenous TDP-43. In particular, overexpression can bypass TDP-43 autoregulatory feedback loops and induce cellular stress responses that influence caspase activation or protein localisation. In addition, all our constructs were C-terminally tagged with GFP to facilitate detection. While this approach enables clear identification of exogenous TDP-43 and quantification, tagging may alter folding, solubility, or aggregation behaviour of TDP-43 and its fragments. Such artefacts could influence cleavage susceptibility or subcellular localisation. Future work using knock-in or endogenously tagged systems will therefore be important to validate these findings under more physiological conditions.

## Conclusion

Our findings reveal a mechanistic axis by which TDP-43 overexpression activates GSK3, promotes caspase-dependent cleavage and fragment accumulation, and contributes to neurotoxicity. Inhibiting GSK3 disrupts this axis, reducing TDP-43 fragment abundance and improving neuronal survival across species and cell types. This work highlights the therapeutic potential of GSK3 inhibition in TDP-43 proteinopathies and opens new avenues for targeting proteostatic imbalances in ALS-FTD. 

## Supplementary Information

Below is the link to the electronic supplementary material.

**TDP-43 knockdown alters GSK3 expression and phosphorylation** (a) Transfection efficiency in SH-SY5Y cells imaged 72 h post-transfection: no siRNA, 25 nM BLOCK-iT™ Alexa Fluor™ Red control siRNA, or 25 nM TARDBP siRNA. Panels show brightfield, Texas Red, and merge channels; BLOCK-iT signal indicates robust siRNA delivery. (b) Representative immunoblot of SH-SY5Y cells transfected with control siRNA or *TARDBP* siRNA for 72 h, probed for inhibitory phosphorylation of GSK3α (Ser21) and GSK3β (Ser9) and for total GSK3α/β; TDP-43 shown to confirm knockdown. (c-e) Quantification of immunoblots: TDP-43 (c),phospho-GSK3α/β normalised to total GSK3α/β (d), total-GSK3α/β (e). Pairwise comparisons: TDP-43, ****(P < 0.0001); phospho-GSK3α, *(P = 0.0307); phospho-GSK3β, *(*P* = 0.0446); total GSK3α, **(*P* = 0.0021); total GSK3β, ** (*P* = 0.0016). for (c) unpaired t test with Welch’s correction, for (d-e) ; two-way ANOVA with Holm–Sidak post-hoc test. Error bars denote mean ± s.e.m. (PNG 2.14 MB)

ESM 1 (TIF 2.95 MB)

**Generation and characterisation of human iPSC-derived forebrainneurons** (a) Schematic of the doxycycline-inducible neurogenin-2 differentiation protocol used to generate forebrain neurons from human induced pluripotent stem cells (iPSCs). (b) Representative immunocytochemistry images at day 14 in vitro showing colocalisation of the neuronal markers MAP2 (green) and NeuN (red) with the nuclear stain DAPI (blue). (c) Quantification of neuronal marker co-localisation; n = 11 wells of cultured neurons. Error bars denote mean ± s.e.m. (PNG 779 KB)

ESM 2 (TIF 1.03 MB)

**GSK3 inhibition influences both the abundance and phosphorylationof GSK3 in iPSC-derived forebrain neurons** (a) Representative immunoblot of iPSC-derived forebrain neurons treated with vehicle (DMSO) or the selective GSK3 inhibitor CHIR99021 for 24 h. (b) Quantification of total GSK3α and GSK3β abundance. Pairwise comparisons: total GSK3α: *(P = 0.0136); total GSK3β: **(P = 0.0038). (c) Quantification of the ratio between phospho-GSK3α/total GSK3α and phospho-GSK3β/total GSK3β. Pairwise comparisons: phospho-GSK3α/total GSK3α: **(P = 0.0022); phospho-GSK3β/total GSK3β: ns (P = 0.1862). (d) Relative normalised expression of GSK3A and GSK3B in SH-SY5Y cells treated with vehicle (DMSO) or CHIR99021 for 24 h. For (b–c) n = 3 biological replicates per condition; one-way ANOVA followed by Holm-Sidak post-hoc test for pairwise comparisons. For (d) n = 3 biological replicates per condition; unpaired two-tailed t-tests with Welch’s correction. Error bars denote mean ± s.e.m. (PNG 371 KB)

ESM 3 (TIF 811 KB)

**Endogenous full-length TDP-43 is unchanged by CHIR99021 and antibody sensitivity is important for the reliable detection of TDP-43 fragments** a) Representative immunoblot of SH-SY5Y cells treated with vehicle (DMSO) or the selective GSK3 inhibitor CHIR99021 for 24 h. (b) Quantification of TDP-43 abundance. Pairwise comparison: DMSO vs. CHIR99021: ns (P = 0.0825); (n = 6 biological replicates per condition). (c) Representative immunoblot of iPSC-derived forebrain neurons treated with DMSO or CHIR99021 for 24 h. (d) Quantification of TDP-43 abundance. Pairwise comparison: DMSO vs. CHIR99021: ns (P = 0.7995); (n = 3 biological replicates per condition). For (b, d): unpaired t test. Error bars denote mean ± s.e.m. (PNG 178 KB)

ESM 4 (TIF 420 KB)

**GSK3 overexpression increases endogenous 35 kDa TDP-43 fragment** (a) Representative immunoblot of SH-SY5Y cells transfected for 24 h with plasmid constructs expressing GFP, GSK3A, GSK3B, or GSK3A + GSK3B. Blots were probed for inhibitory phosphorylation of GSK3α (Ser21) and GSK3β (Ser9), total GSK3α and total GSK3β, TDP-43, and Cyclophilin (loading control). An additional over-exposed TDP-43 panel is provided to aid visualisation of the low-abundance 35 kDa TDP-43 species. (b–d) Quantification of endogenous TDP-43 immunoblot bands: (b) full-length TDP-43: pairwise comparisons vs GFP vs GSK3A: ns (*P* = 0.1349); GSK3B: ns (*P* = 0.5559); GSK3A + GSK3B: ns (*P* = 0.5559); (c) 35 kDa TDP-43 normalised to Cyclophilin: pairwise comparisons vs GFP vs GSK3A: ns (P = 0.6338); GSK3B: ns (*P* = 0.1393); GSK3A + GSK3B: ns (*P* = 0.0581); (d) 35 kDa TDP-43 expressed relative to full-length TDP-43: pairwise comparisons vs GFP vs GSK3A: * (*P* = 0.0462); GSK3B: * (*P* = 0.0462); GSK3A + GSK3B: * (*P* = 0.0462). For (b-d) (n = 3 biological replicates per condition; one-way ANOVA followed by Holm-Sidak post-hoc test for pairwise comparisons. Error bars denote mean ± s.e.m. (PNG 500 KB)

ESM 5 (TIF 895 KB)

**Uncropped Western blot images** Uncropped Western blot images corresponding to: a. Figure 1b - SH-SY5Y cells transfected with expression constructs for GFP and GFP tagged: TDP-43^N-Del^, TDP-43^WT^ or TDP-43^Q331K^ for 24 hours with and without treatment with the pan caspase inhibitor Q-VD-OPh. b. Figure 2a - SH-SY5Y cells transfected with expression constructs for GFP and GFP tagged: TDP-43^N-Del^, TDP-43^WT^, TDP-43^N-Del^, TDP-43^Q331K^ or TDP-43D89E for 24 hours with and without treatment with the potent pro-apoptotic caspase activator staurosporine. c. Figure 3a - SH-SY5Y cells transfected with expression constructs for GFP and GFP tagged: TDP-43^N-Del^, TDP-43^WT^ or TDP-43^Q331K^ for 24 hours and blotted for phospho-GSK3α/β (Ser21/9) and total GSK3α/β. d. Figure 3c - SH-SY5Y cells with and without treatment with the small molecule GSK3 inhibitor CHIR99021 for 24 hours. e. Figure 4a - SH-SY5Y cells transfected with expression constructs for GFP and GFP tagged: TDP-43^N-Del^, TDP-43^WT^ or TDP-43^Q331K^ for 24 hours and treated with the GSK3 inhibitors CHIR99021 (CHIR) or AZD1080 (AZD). Figure 4e - SH-SY5Y cells treated with CHIR99021 (24 h) and probed for endogenous TDP-43, with an overexposed panel to visualise the low abundance 35 kDa fragment. f. Supplementary Figure 1b - SH-SY5Y cells transfected with control siRNA or TARDBP siRNA for 72 h, probed for inhibitory phosphorylation of GSK3α (Ser21) and GSK3β (Ser9) and for total GSK3α/β. g. Supplementary Figure 3a - iPSC-derived forebrain neurons treated with vehicle (DMSO) or the selective GSK3 inhibitor CHIR99021 for 24 h. h. Supplementary Figure 4a - SH-SY5Y cells treated with vehicle (DMSO) or the selective GSK3 inhibitor CHIR99021 for 24 h. Supplementary Figure 4c - iPSC-derived forebrain neurons treated with DMSO or CHIR99021 for 24 h. i. SH-SY5Y cells transfected for 24 h with plasmid constructs expressing GFP, GSK3A, GSK3B, or GSK3A + GSK3B. Blots were probed for inhibitory phosphorylation of GSK3α (Ser21) and GSK3β (Ser9), total GSK3α and total GSK3β, TDP-43, and Cyclophilin (loading control). (PNG 1.11 MB)

ESM 6 (TIF 2.67 MB)

**Antibodies and compounds used in this study** Uncropped Western blot images Uncropped Western blot images corresponding to: a. Figure 1b - SH-SY5Y cells transfected with expression constructs for GFP and GFP tagged: TDP-43^N-Del^, TDP-43^WT^ or TDP-43^Q331K^ for 24 hours with and without treatment with the pan caspase inhibitor Q-VD-OPh. b. Figure 2a - SH-SY5Y cells transfected with expression constructs for GFP and GFP tagged: TDP-43^N-Del^, TDP-43^WT^, TDP-43^N-Del^, TDP-43^Q331K^ or TDP-43^D89E^ for 24 hours with and without treatment with the potent pro-apoptotic caspase activator staurosporine. c. Figure 3a - SH-SY5Y cells transfected with expression constructs for GFP and GFP tagged: TDP-43^N-Del^, TDP-43^WT^ or TDP-43^Q331K^ for 24 hours and blotted for phospho-GSK3α/β (Ser21/9) and total GSK3α/β. d. Figure 3c - SH-SY5Y cells with and without treatment with the small molecule GSK3 inhibitor CHIR99021 for 24 hours. e. Figure 4a - SH-SY5Y cells transfected with expression constructs for GFP and GFP tagged: TDP-43^N-Del^, TDP-43^WT^ or TDP-43^Q331K^ for 24 hours and treated with the GSK3 inhibitors CHIR99021 (CHIR) or AZD1080 (AZD). Figure 4e - SH-SY5Y cells treated with CHIR99021 (24 h) and probed for endogenous TDP-43, with an overexposed panel to visualise the low abundance 35 kDa fragment. f. Supplementary Figure 1b - SH-SY5Y cells transfected with control siRNA or TARDBP siRNA for 72 h, probed for inhibitory phosphorylation of GSK3α (Ser21) and GSK3β (Ser9) and for total GSK3α/β. g. Supplementary Figure 3a - iPSC-derived forebrain neurons treated with vehicle (DMSO) or the selective GSK3 inhibitor CHIR99021 for 24 h. h. Supplementary Figure 4a - SH-SY5Y cells treated with vehicle (DMSO) or the selective GSK3 inhibitor CHIR99021 for 24 h. Supplementary Figure 4c - iPSC-derived forebrain neurons treated with DMSO or CHIR99021 for 24 h. i. SH-SY5Y cells transfected for 24 h with plasmid constructs expressing GFP, GSK3A, GSK3B, or GSK3A + GSK3B. Blots were probed for inhibitory phosphorylation of GSK3α (Ser21) and GSK3β (Ser9), total GSK3α and total GSK3β, TDP-43, and Cyclophilin (loading control). (PNG 243 KB)

ESM 7 (TIF 310 KB)

## Data Availability

The datasets used and/or analysed during the current study are available from the corresponding author on reasonable request.

## Abbreviations

ALS-FTD: Amyotrophic lateral sclerosis-frontotemporal dementia
TDP-43: 43-KDa TAR DNA-binding protein
GSK3: Glycogen synthase kinase-3
ALS: Amyotrophic lateral sclerosis
FTD: Frontotemporal dementia
LATE: Limbic-predominant age-related TDP-43 encephalopathy
DIV: Days in vitro
ROIs: Regions of interest
NGN2: Neurogenin-2
iPSC: Induced pluripotent stem cell
UTRs: Untranslated regions
RAN: Repeat-associated non-AUG
VAPB: Vesicle-associated membrane protein-associated protein-B
