# Supplementary figures and images for: Inhibiting Glycogen Synthase Kinase 3 Suppresses TDP-43-Mediated Neurotoxicity in a Caspase-Dependent Manner
Source: Mol Neurobiol. 2026 Jan 17;63(1):370. doi: 10.1007/s12035-026-05675-5 (PMC12812098; doi:10.1007/s12035-026-05675-5)

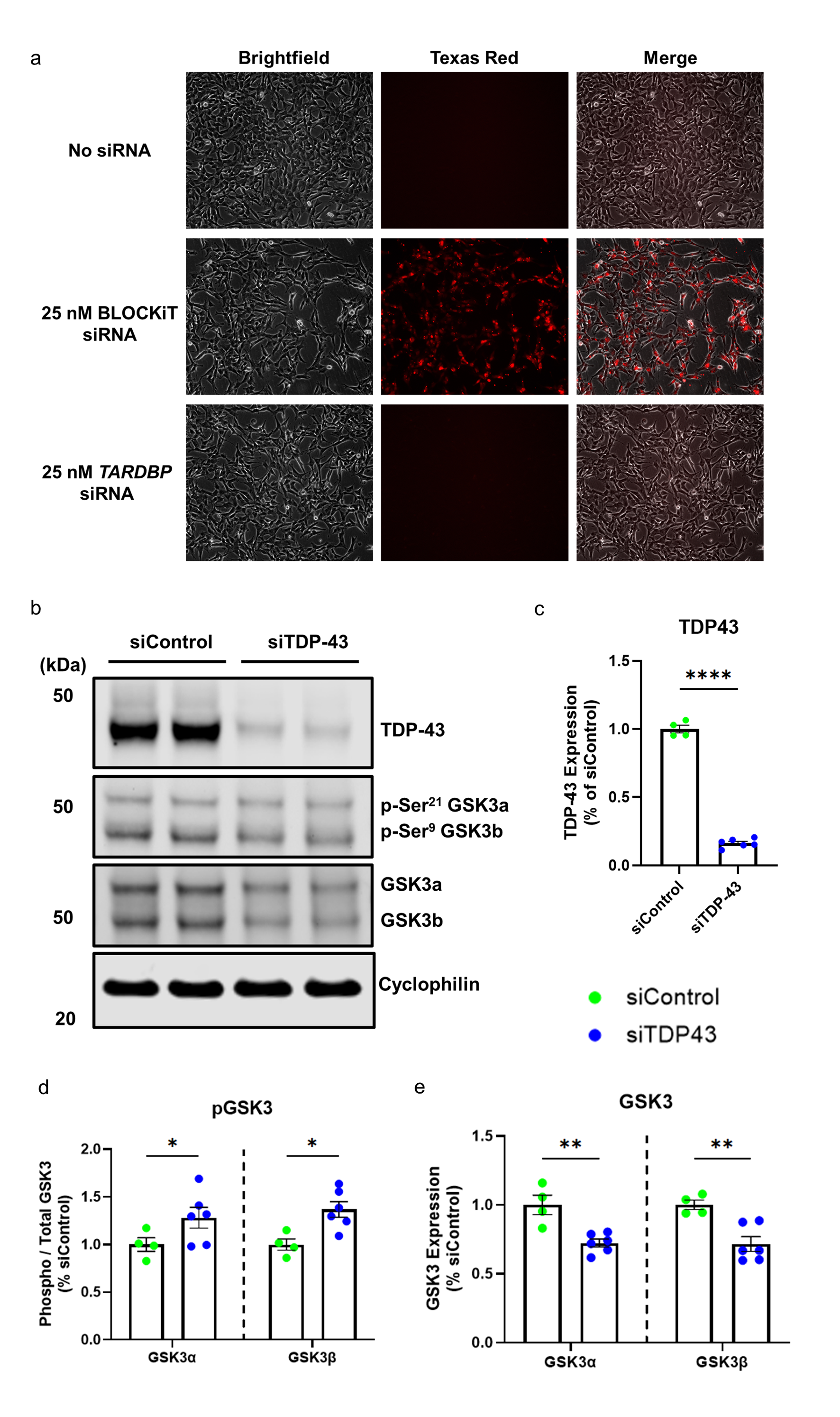

Supplement: Supplementary file 1 — (PNG 2.14 MB) [file 12035_2026_5675_Fig8_ESM.png]

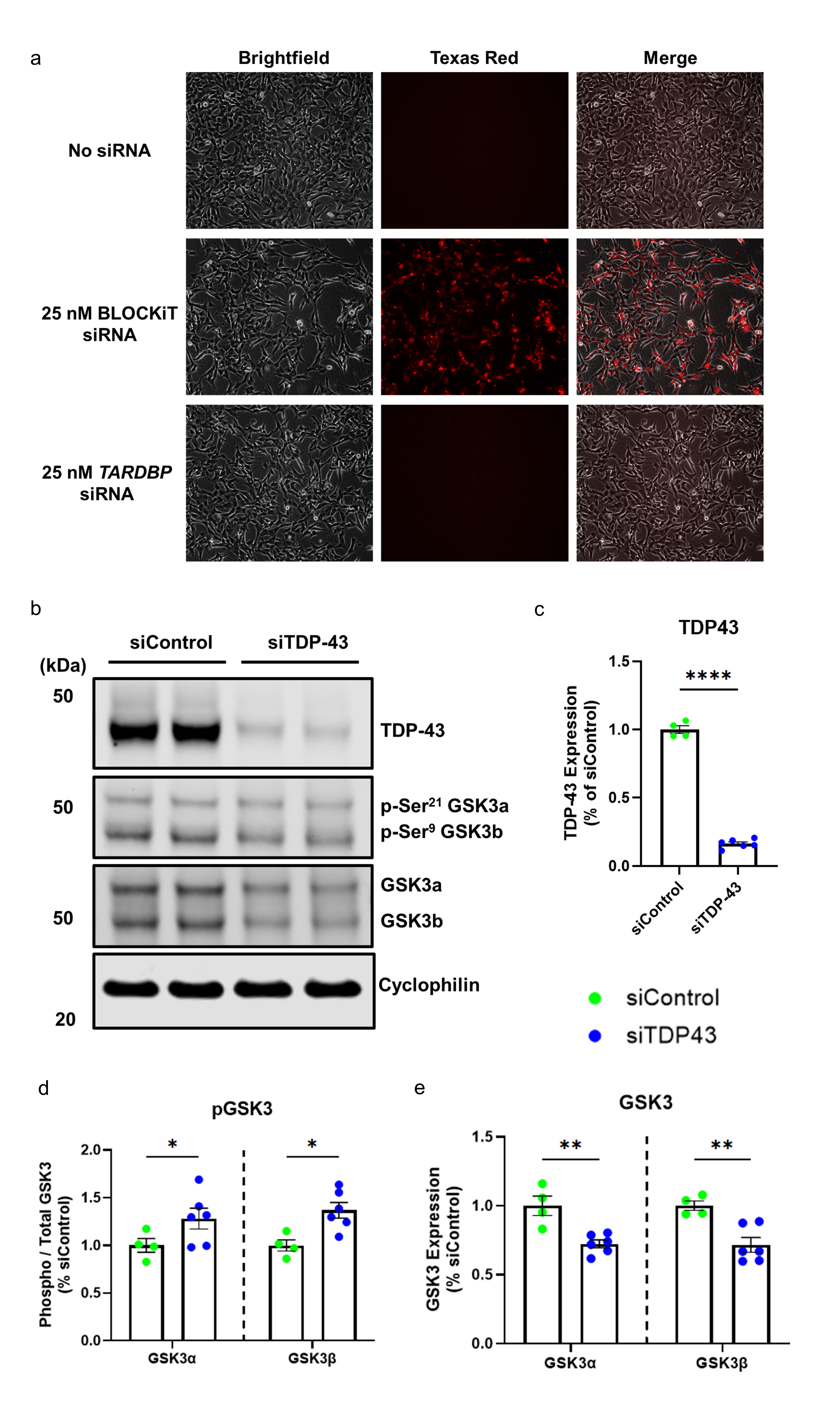

Supplement: Supplementary file 2 — (TIF 2.95 MB) [file 12035_2026_5675_MOESM1_ESM.tif]

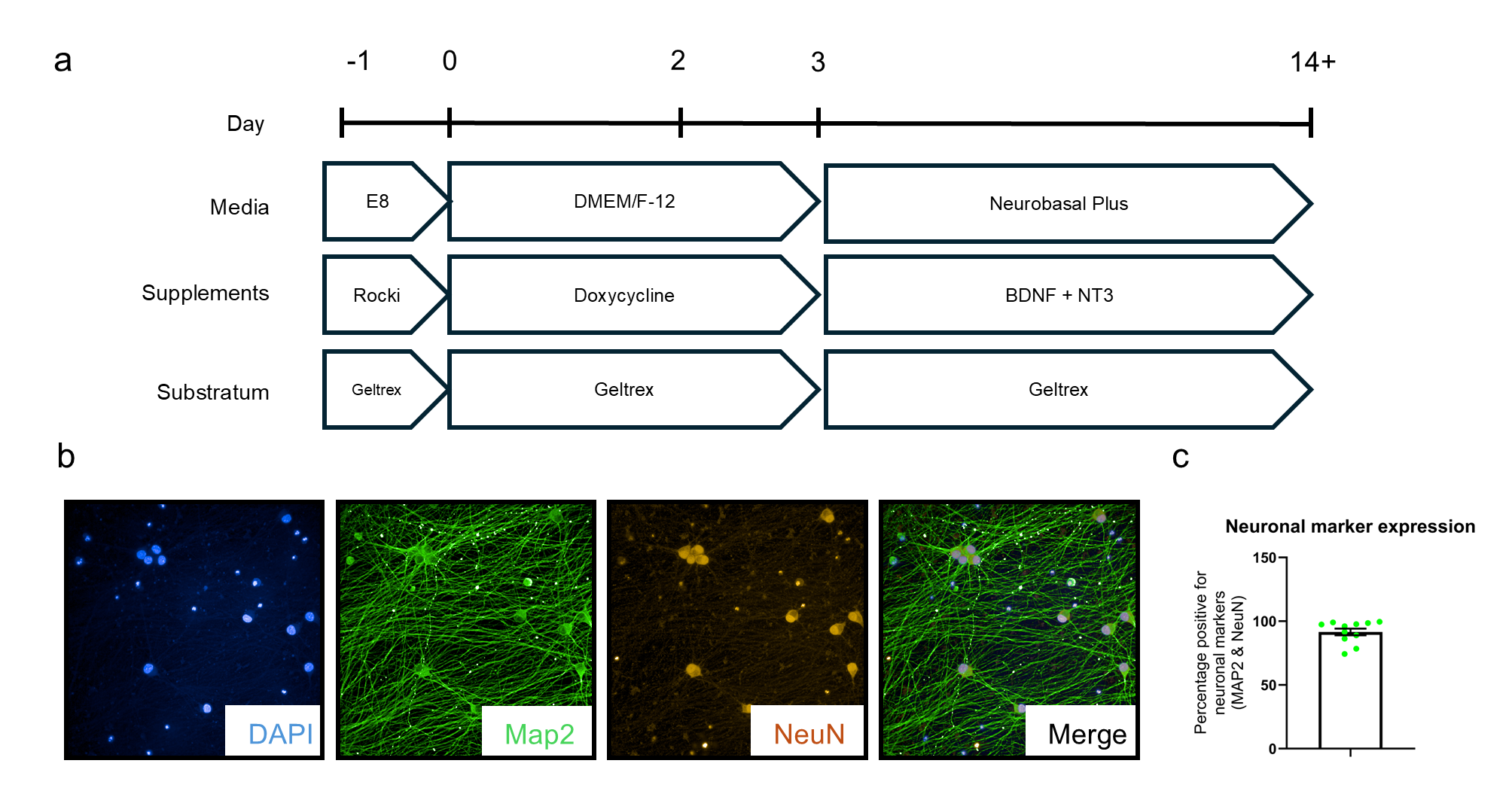

Supplement: Supplementary file 3 — (PNG 779 KB) [file 12035_2026_5675_Fig9_ESM.png]

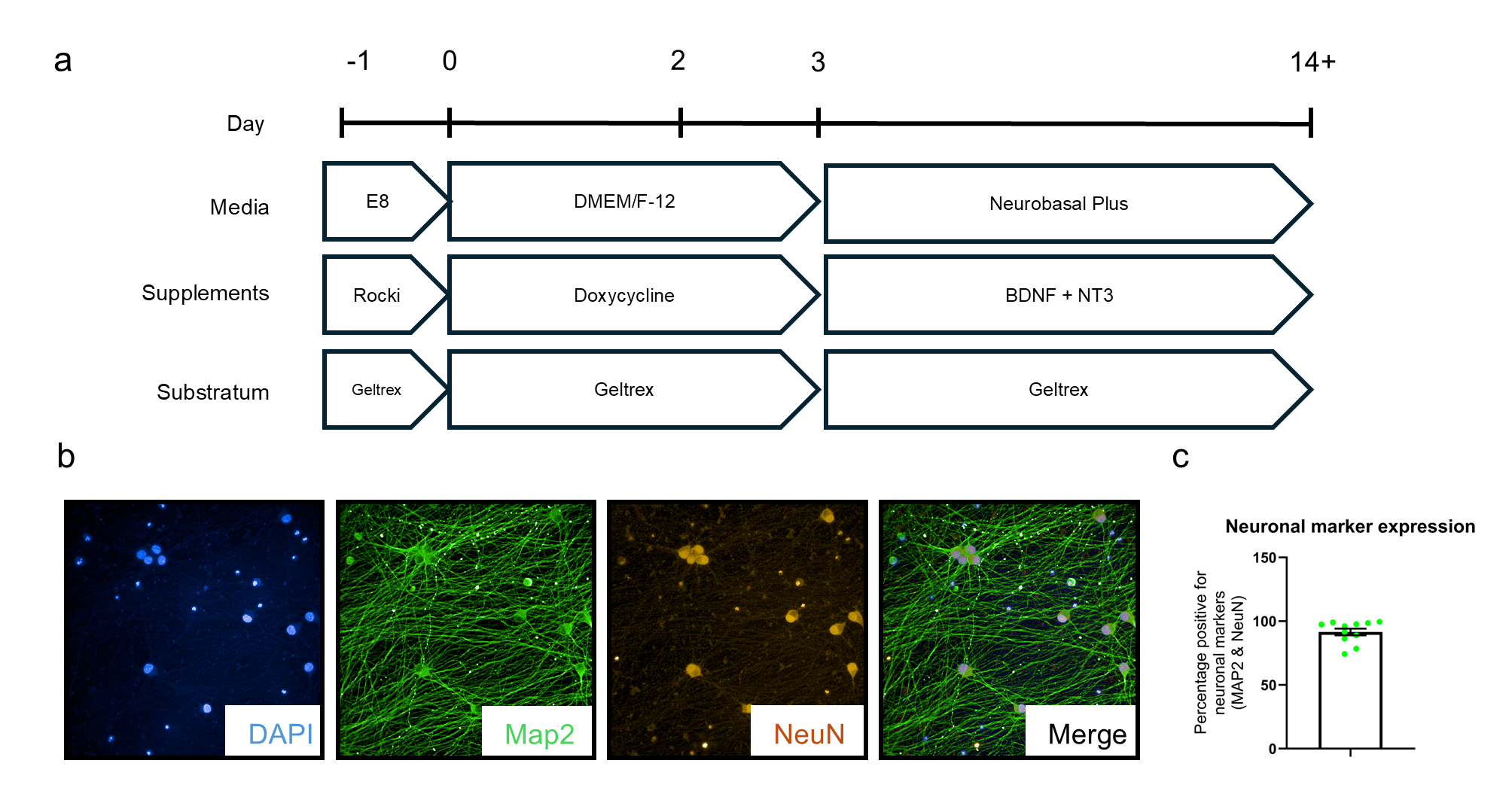

Supplement: Supplementary file 4 — (TIF 2.95 MB) [file 12035_2026_5675_MOESM2_ESM.tif]

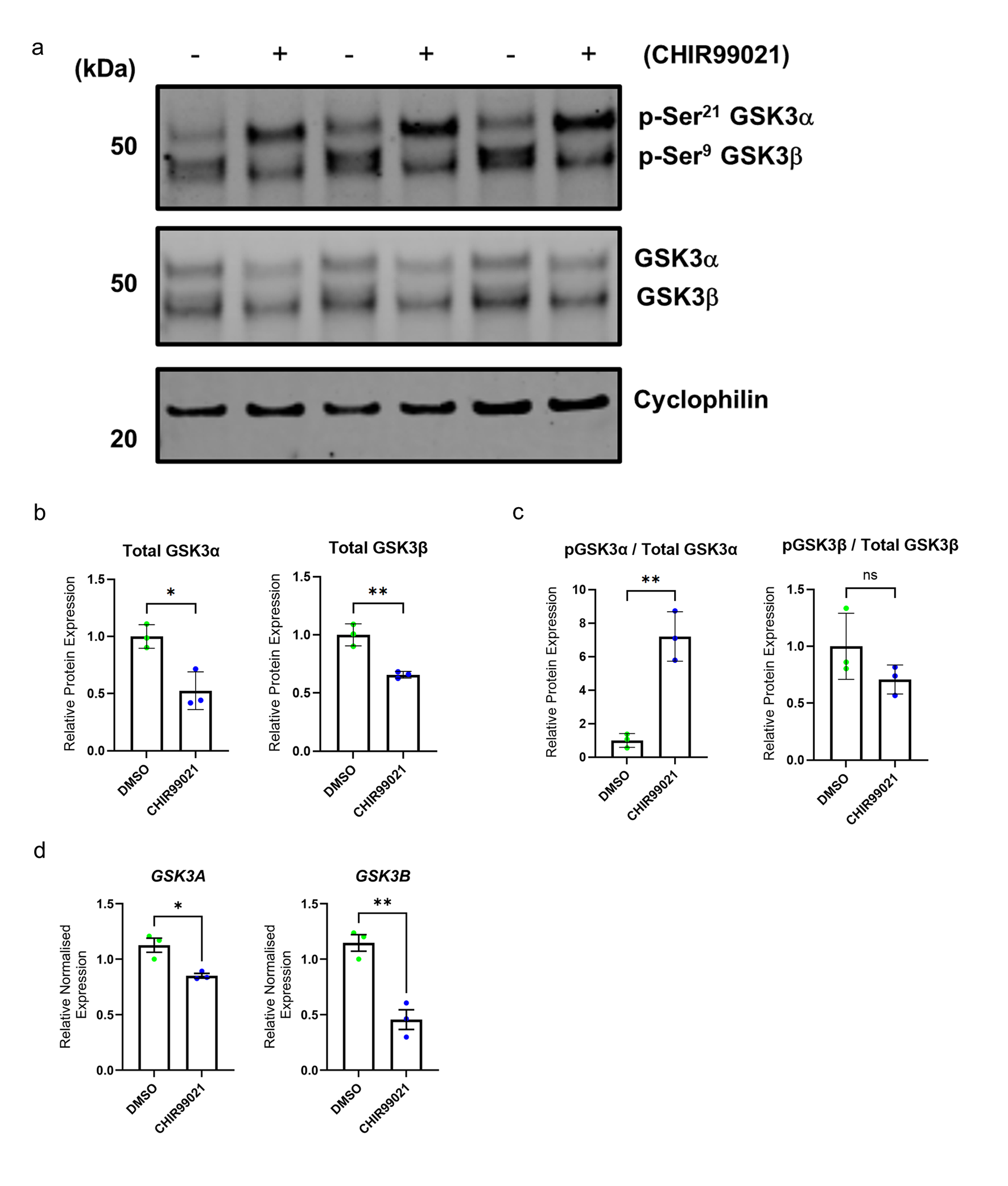

Supplement: Supplementary file 5 — (PNG 371 KB) [file 12035_2026_5675_Fig10_ESM.png]

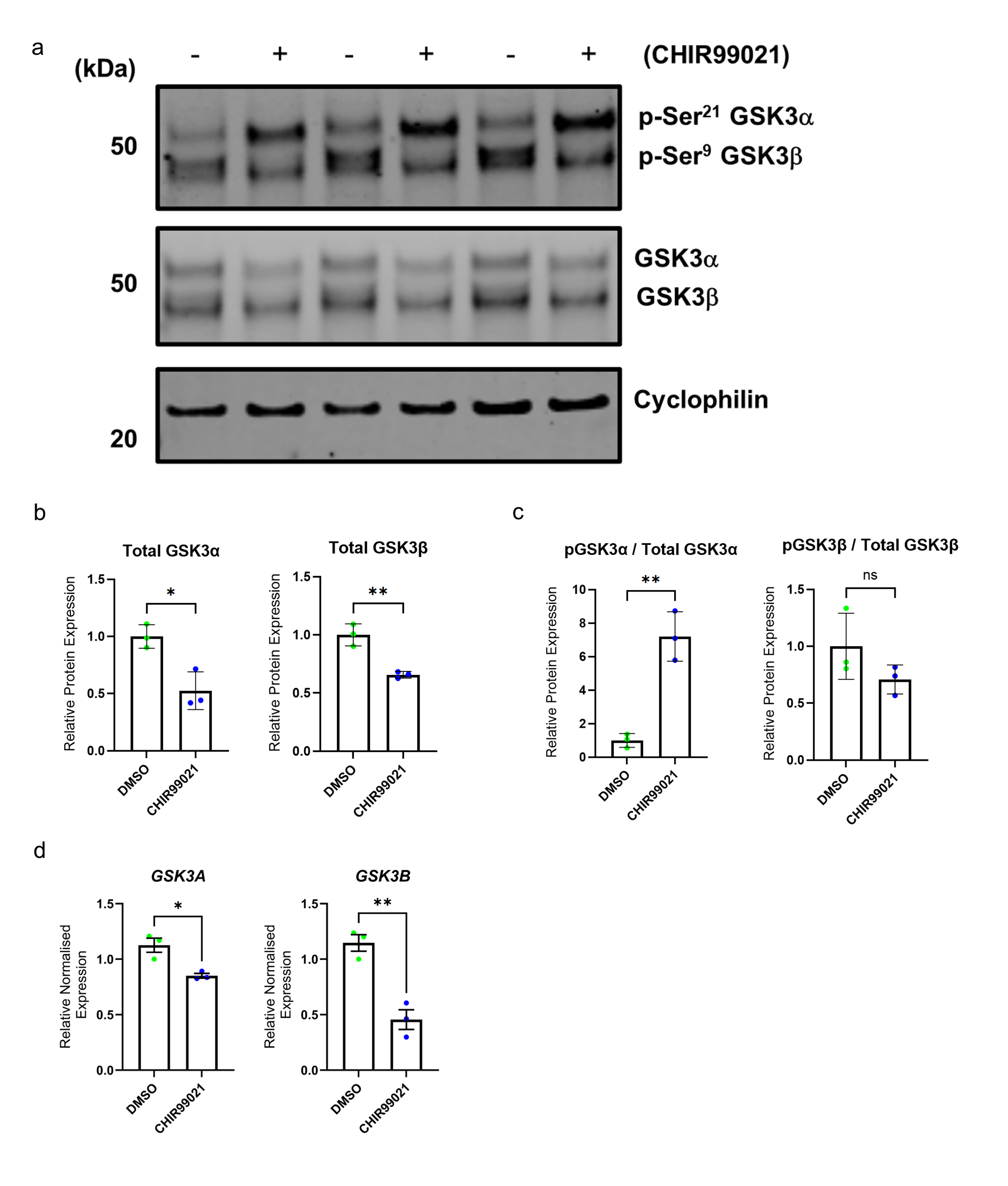

Supplement: Supplementary file 6 — (TIF 811 KB) [file 12035_2026_5675_MOESM3_ESM.tif]

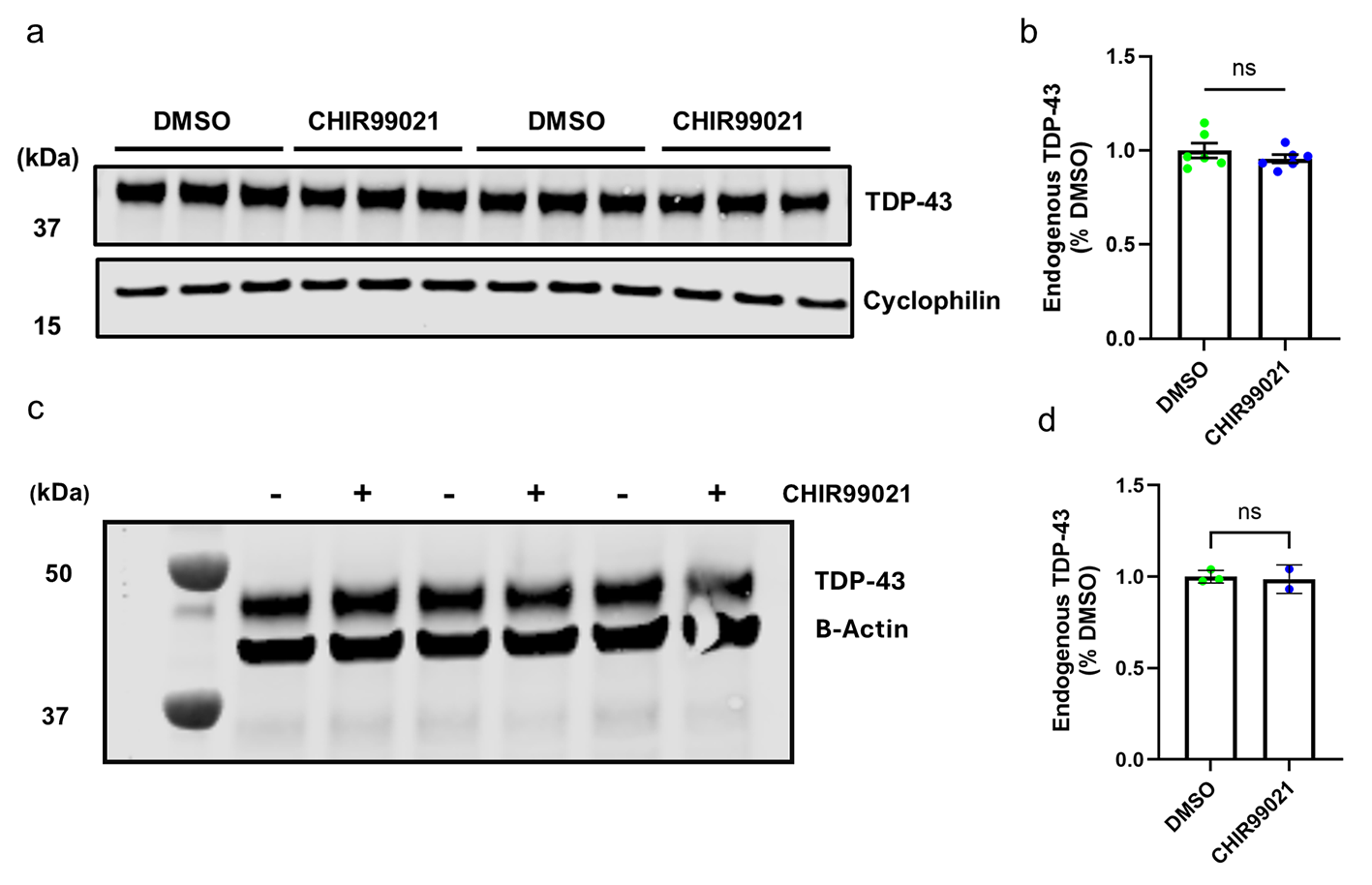

Supplement: Supplementary file 7 — (PNG 178 KB) [file 12035_2026_5675_Fig11_ESM.png]

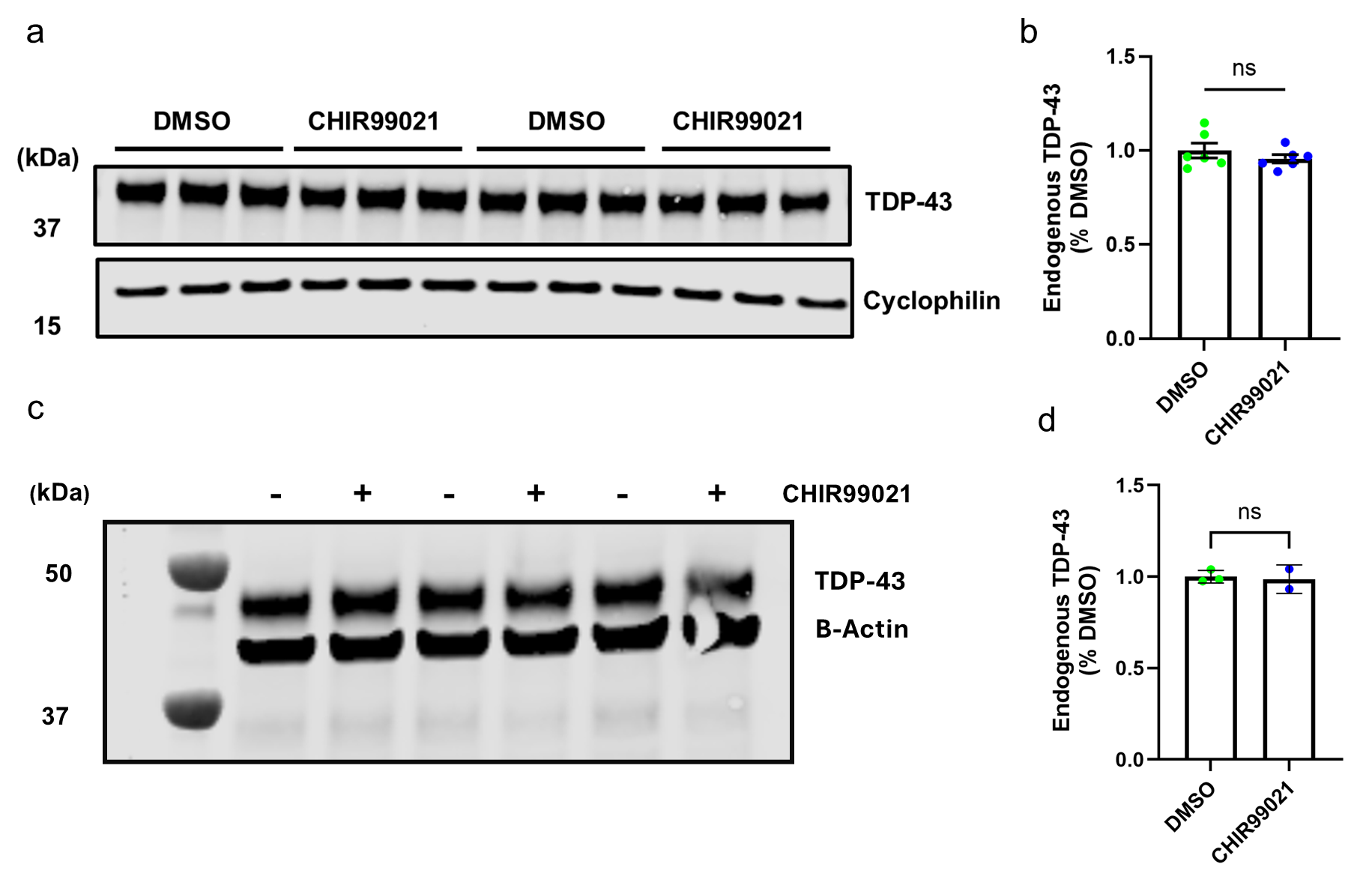

Supplement: Supplementary file 8 — (TIF 420 KB) [file 12035_2026_5675_MOESM4_ESM.tif]

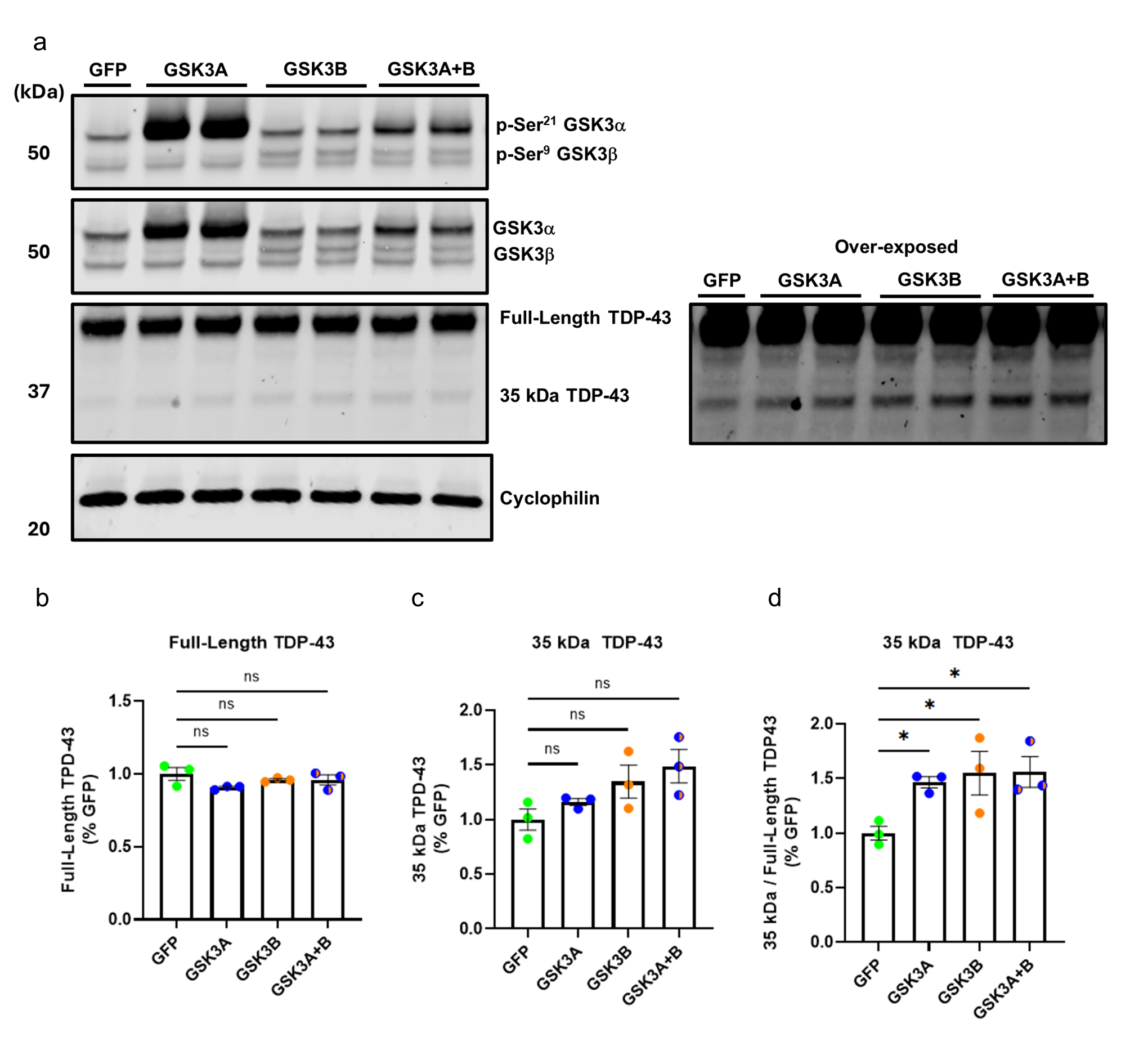

Supplement: Supplementary file 9 — (PNG 500 KB) [file 12035_2026_5675_Fig12_ESM.png]

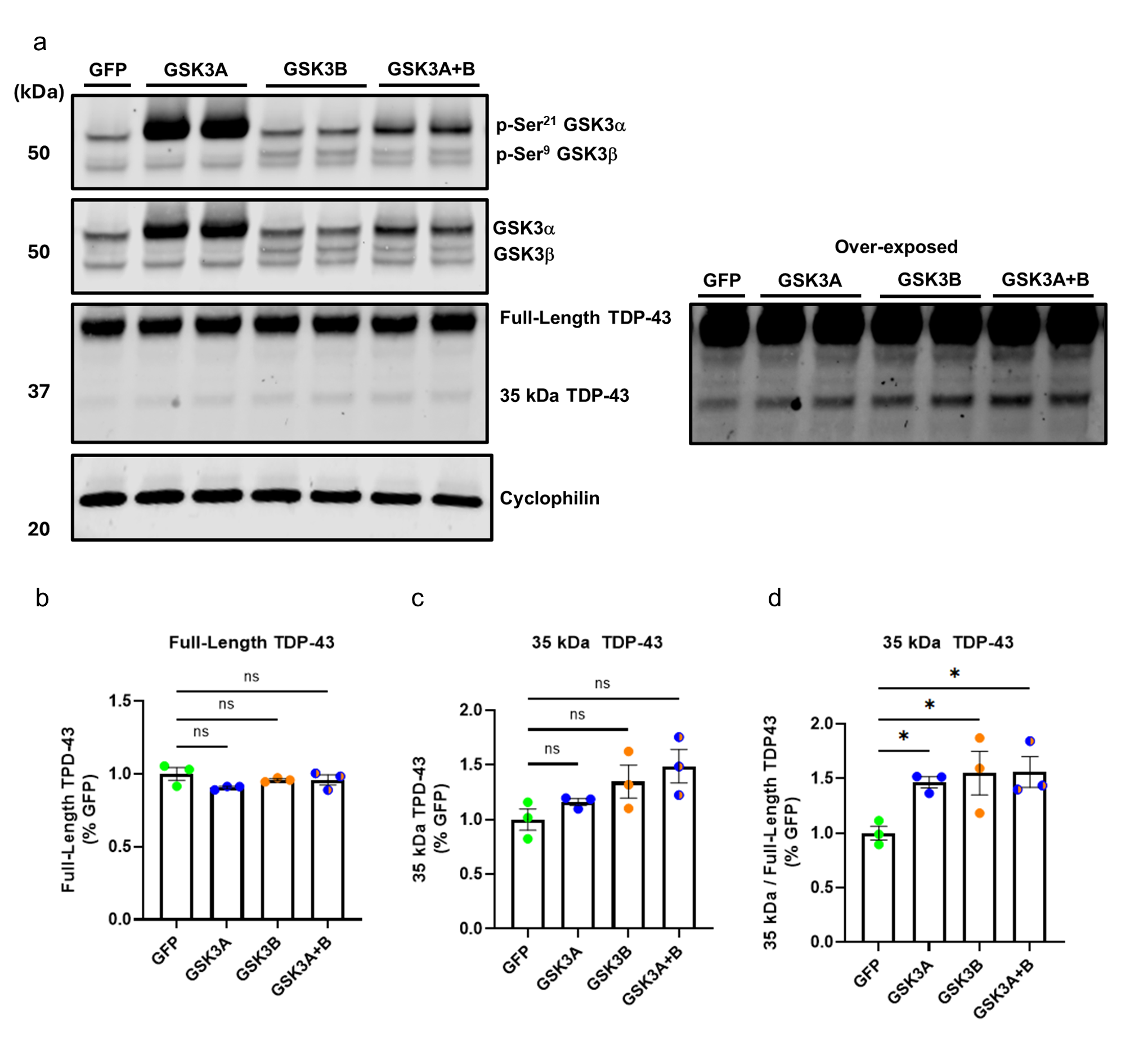

Supplement: Supplementary file 10 — (TIF 895 KB) [file 12035_2026_5675_MOESM5_ESM.tif]

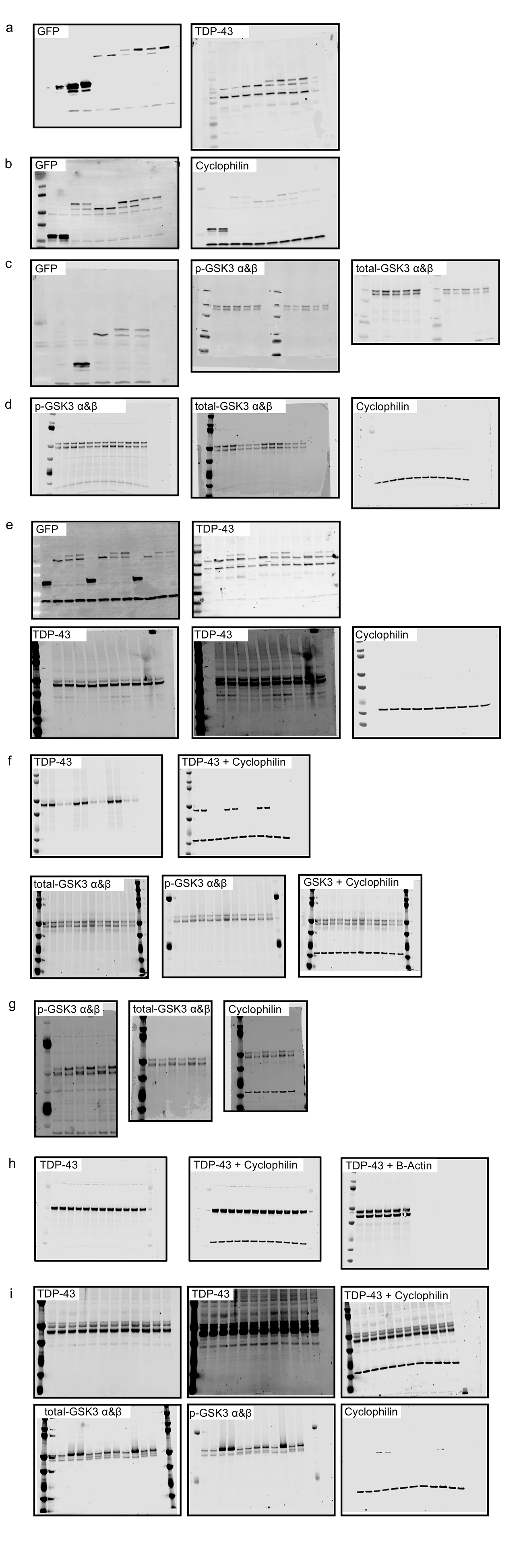

Supplement: Supplementary file 11 — (PNG 1.11 MB) [file 12035_2026_5675_Fig13_ESM.png]

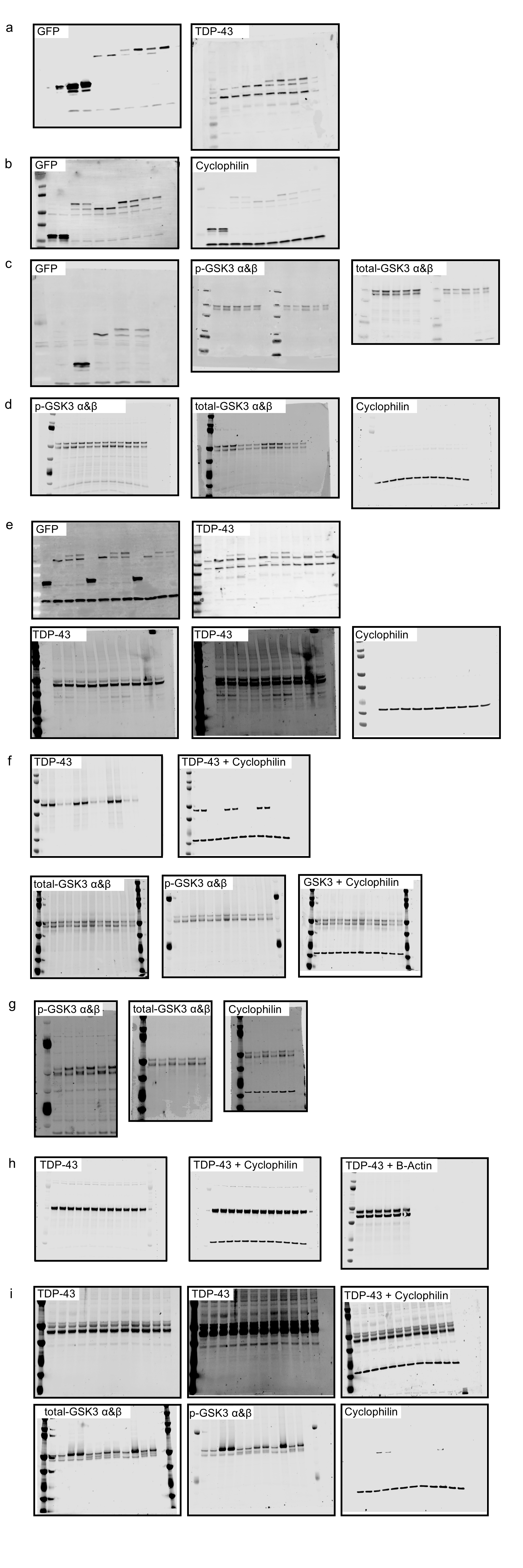

Supplement: Supplementary file 12 — (TIF 2.67 MB) [file 12035_2026_5675_MOESM6_ESM.tif]

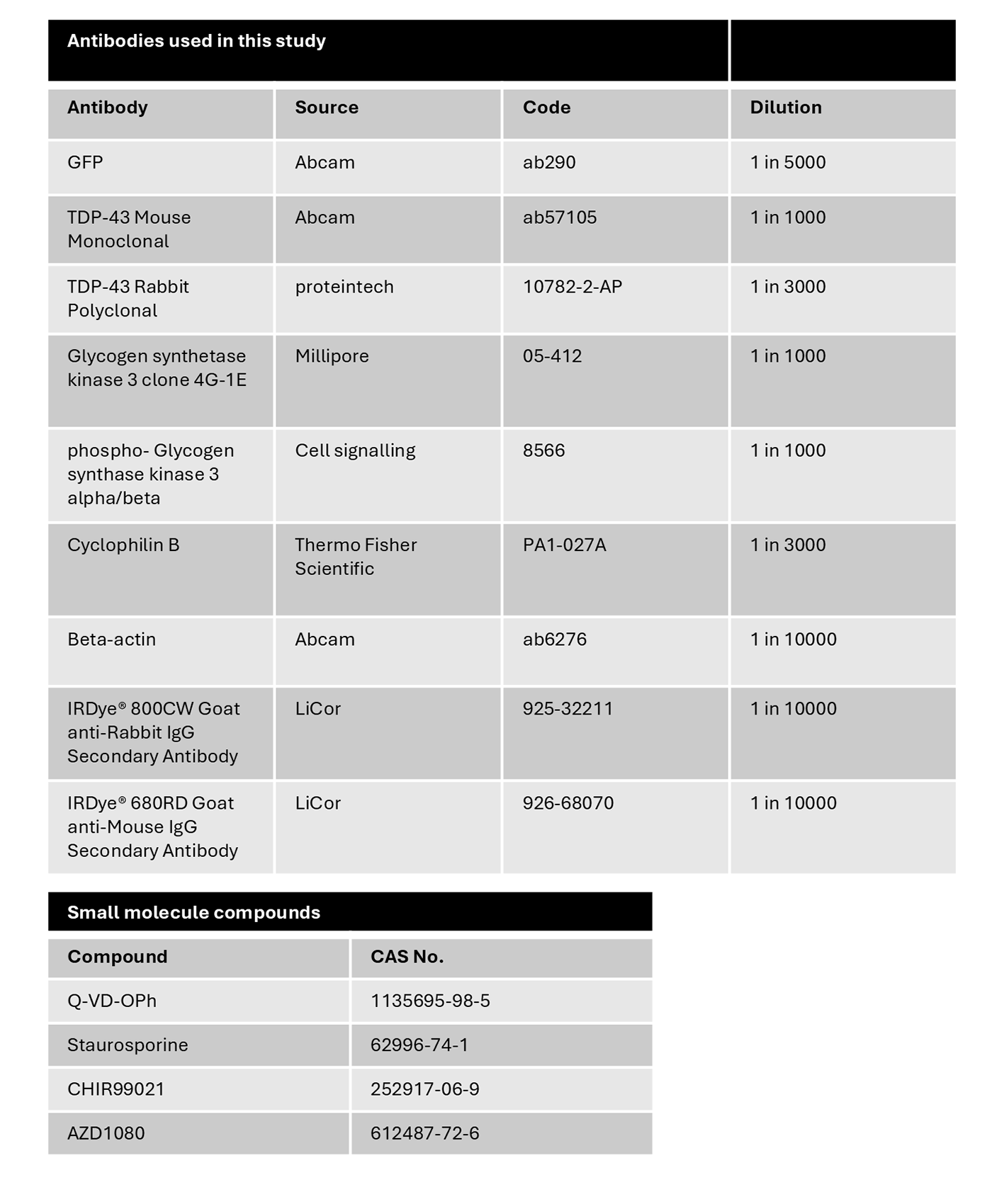

Supplement: Supplementary file 13 — (PNG 243 KB) [file 12035_2026_5675_Fig14_ESM.png]

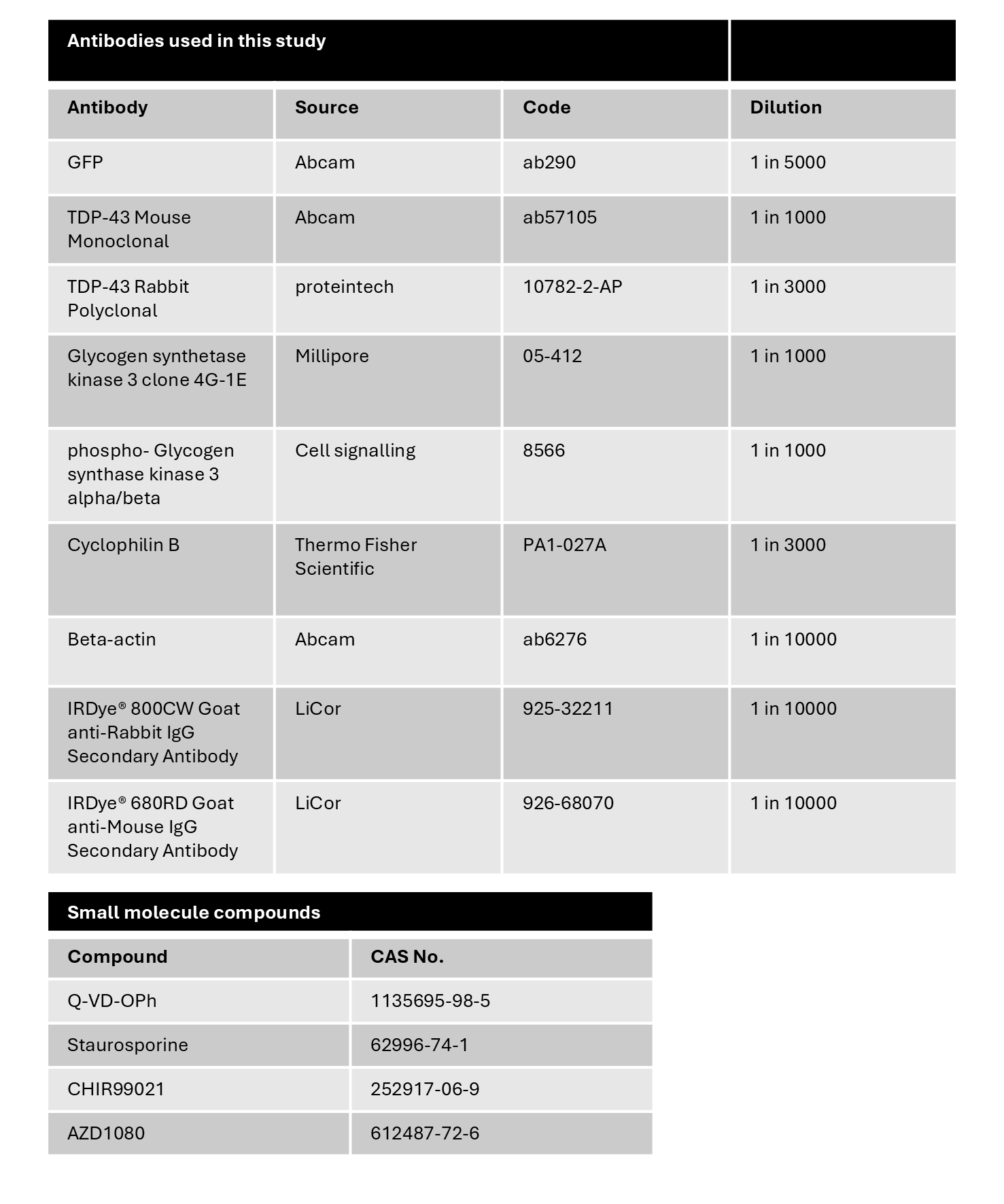

Supplement: Supplementary file 14 — (TIF 310 KB) [file 12035_2026_5675_MOESM7_ESM.tif]
